# A Peer-Led, Nurse-Involved Blended Online and Offline Peer Support Program (PNO2PSP) for Psychosocial Adjustment in Young- to Middle-Aged Patients With Breast Cancer: Cluster Randomized Clinical Trial

**DOI:** 10.2196/86097

**Published:** 2026-04-17

**Authors:** Yiheng Zhang, Haiyan He, Ye Zhang, Youxian Zhong, Chunhong Luo, Juanjuan Chen, Lili Chen, Ni Zhang, Shihao Sun, Baoyi Zhang, Jia Fang, Jingwen Yan, Meifen Zhang

**Affiliations:** 1 School of Nursing, Jinan University, Guangzhou, China; 2 The First Affiliated Hospital, Jinan University, Guangzhou, Guangdong, China; 3 Department of Nursing, Sun Yat-sen Memorial Hospital, Sun Yat-sen University, Guangzhou, China; 4 School of Nursing, Sun Yat-sen University, No.74 Zhongshan 2nd Road, Yuexiu District, Guangzhou, Guangdong Province, 510080, China, 86 13416225022

**Keywords:** young to middle-aged patients with breast cancer, psychosocial adjustment, peer support, blended online-offline intervention, cluster randomized clinical trial

## Abstract

**Background:**

Young- to middle-aged patients with breast cancer face significant psychosocial challenges. Existing interventions often lack comprehensiveness, timely initiation, and tailoring specific to this population’s unique needs.

**Objective:**

This study aimed to evaluate the impact of a peer-led, nurse-involved, blended online and offline peer support intervention program (PNO2PSP) on psychosocial adjustment in young- to middle-aged patients with breast cancer.

**Methods:**

The effectiveness of PNO2PSP was evaluated through a single-center cluster randomized controlled trial involving 70 newly diagnosed young- to middle-aged patients with breast cancer (35 in each group). The intervention group received an 8-week PNO2PSP in addition to routine care. Psychosocial adjustment, self-efficacy, social support, and coping modes were assessed presurgery and at 4, 8, and 12 weeks post surgery. Generalized estimating equations were used for intention-to-treat analyses. In-depth interviews with 9 participants explored their experiences.

**Results:**

Compared to the control group, the intervention group demonstrated significantly lower psychosocial adjustment scores at 8 weeks (T2; Wald *χ²*_1_=14.96; *P*<.001) and 12 weeks (T3; Wald *χ²*_1_=7.49; *P*=.006); social support was higher at 8 weeks (T2; Wald *χ²*_1_=7.65; *P*=.006). Confrontation coping scores were higher at T2 (Wald *χ²*_1_=5.46; *P*=.02), T3 (Wald *χ²*_1_=10.04; *P*=.002), while avoidance coping scores were lower at T1 (Wald *χ²*_1_=8.24; *P*=.004), T2 (Wald *χ²*_1_=7.45; *P*=.006), and T3 (Wald *χ²*_1_=5.70; *P*=.02). Qualitative findings supported these quantitative results, highlighting the program’s role in facilitating psychosocial adjustment, providing vital support, boosting treatment confidence, and fostering positive coping.

**Conclusions:**

The PNO2PSP effectively improved psychosocial adjustment, social support, and positive coping in young- to middle-aged patients with breast cancer. Its scientifically validated, feasible, and patient-centered design supports its recommendation for wider clinical implementation, with continued training for peer supporters and sustained delivery of peer support.

## Introduction

Breast cancer remains one of the most common malignancies among women and a major threat to their health. In 2022, approximately 2.3 million new cases were reported globally, accounting for 23.8% of all female cancer diagnoses. Notably, young- to middle-aged women (aged 18‐59 years) comprised 53.9% of these cases [1]. The diagnosis of breast cancer is a life-altering event that brings multifaceted psychosocial challenges, particularly for young- to middle-aged women [2]. These challenges include fears of mortality associated with the diagnosis, body image disturbances caused by mastectomy, treatment side effects such as alopecia and sexual dysfunction, as well as significant changes in family and social roles, such as reduced work capacity and limitations in household activities [3-5]. These issues underscore the significant psychosocial adjustment challenges faced by young- to middle-aged patients with breast cancer.

Psychosocial adjustment refers to the adaptation status of individuals experiencing emotional and social problems caused by illness and treatment [6]. Poor psychosocial adjustment not only hampers patients’ return to normal life and recovery but may also compromise immune function, potentially worsening disease prognosis [7-9]. For young- to middle-aged women, the challenges of psychosocial adjustment are even more pronounced due to their substantial family and societal responsibilities, including career development and child-rearing [9-11]. Studies have shown that younger patients with breast cancer often experience more severe long-term anxiety, depression, loneliness [12], physical dysfunction [9,13], changes in sexual relationships [4,14], fertility concerns [15], and difficulties in returning to work and social participation [5], all of which exacerbate their psychosocial maladjustment. Compared with older patients, young- and middle-aged women face particularly prominent challenges related to sexual function and fertility [16]. However, these concerns have not been sufficiently emphasized in previous research.

While various interventions have been developed to address psychosocial adjustment issues in patients with breast cancer, such as cognitive-behavioral interventions [17], mindfulness-based interventions [18], psychoeducation [19], spousal support [20], and multidisciplinary psychosocial interventions [21,22], these approaches have demonstrated some effectiveness, but also have limitations. First, most interventions focus solely on either psychological or social support without comprehensively addressing psychosocial adjustment. Second, insufficient evidence linking intervention targets to outcomes often results in suboptimal effectiveness and inefficient resource usage. Third, many interventions are initiated only after treatment has begun, lacking the timeliness of early intervention. Finally, there is a notable lack of targeted interventions specifically designed for young- to middle-aged patients with breast cancer.

Previous research by our team identified social support, self-efficacy, and coping modes as key predictors of psychosocial adjustment in this population [6,23]. These factors can serve as crucial targets for developing effective interventions. Within the concept of peer support, it is evident that peer support can improve health outcomes by augmenting social networks, enhancing effective coping strategies, and increasing self-efficacy [24]. Thus, this study adopts peer support as the primary intervention approach to improve psychosocial adjustment among young- to middle-aged patients with breast cancer. Peer support refers to the approach whereby individuals with the same disease or condition meet to exchange information, share experiences, and encourage or help each other to overcome difficulties [25]. Although peer-support interventions for patients with breast cancer have been developed, most focus on outcomes such as negative emotions or quality of life [26-29]. However, these interventions often lack clear mechanisms, theoretical grounding, and differentiation in content, while insufficiently addressing the specific needs of young- to middle-aged women.

Previous research has summarized 6 modes of peer intervention: one-on-one face-to-face, one-on-one telephone, one-on-one online, group face-to-face, group telephone, and group online settings [25]. Peer support is primarily categorized into group-based and one-on-one formats. While one-on-one support pairs a patient with a specific peer, the process is susceptible to compatibility issues [30]; inappropriate pairing may lead to intervention failure and require higher competency from the peer. In contrast, group-based support typically consists of 3 or more members, fostering a collaborative environment for sharing knowledge and experience while providing emotional, social, or substantive assistance [31].

Peer training is a critical component in consolidating the effectiveness of peer support, as it alleviates the psychological pressure and burden on peers while maximizing the safety and quality of the intervention [32-34]. Consequently, all peer supporters in this study underwent unified screening and standardized training. Furthermore, to address concerns regarding the accessibility of peer support and the reliability of information within groups, health care professionals were involved to provide specialized information and standardize group procedures, thereby preventing interpersonal conflicts and public disputes [34,35]. Professional involvement is thus essential to ensure informational accuracy. Therefore, this study was designed as a peer-led, nurse-involved intervention.

Regarding the delivery method, face-to-face interventions have been shown to facilitate deeper communication; however, long-term offline implementation is often constrained by distance and costs, leading to patient attrition and sustainability challenges [36,37]. With the diversification of social interaction, online support groups have become increasingly prevalent, offering temporal and spatial flexibility that facilitates broader dissemination [38]. To balance intervention efficacy with participant engagement, a blended online and offline model was adopted: offline sessions were conducted during hospitalization to build rapport among members, followed by continuous online support after discharge. Given this context, this study aims to develop and evaluate a peer-led, nurse-involved blended online and offline peer support program (PNO2PSP) tailored to young- to middle-aged patients with breast cancer. The goal is to provide an innovative and effective pathway to enhance psychosocial adjustment in young- to middle-aged patients with breast cancer.

## Methods

### Research Design Overview

This study used a single-center cluster randomized controlled trial (CRCT) to evaluate the effectiveness of PNO2PSP. The control group received routine care, while the intervention group received the comprehensive PNO2PSP intervention.

### Participants

Convenience sampling was used to recruit newly diagnosed young- to middle-aged patients with breast cancer who were admitted for surgical treatment to the Department of Breast Surgery, Sun Yat-sen Memorial Hospital (Textbox 1).

Textbox 1. **Inclusion and exclusion criteria.**

**Inclusion criteria:**

- Pathologically diagnosed with breast cancer (stage I, II, or III).
- Aged 18‐59 years.
- Aware of their diagnosis.
- Voluntary participation with informed consent.
- Able to independently complete questionnaires.

**Exclusion criteria:**

- Receiving neoadjuvant chemotherapy.
- Communication barriers due to sensory impairments.
- Other life-threatening conditions or psychiatric disorders.
- History of recurrence, metastasis, or other malignancies.

### Sample Size

Based on the sample size calculation formula for experimental studies, using a 2-tailed test, the primary outcome measure of this study was the score on the psychosocial adjustment scale. Referencing previous research [39], the sample size per group was calculated to be at least 18 participants. To account for potential dropouts and other factors, a 15% increase was applied, resulting in 21 participants per group, for a total of 42 participants.

To further accommodate the cluster-randomized design at the enrollment week level, reference was made to previous longitudinal psychosocial intervention studies, which reported a design effect (DE) of 1.472 [40]. By applying this DE to the initial estimate (42×1.472=62.04), it was determined that a minimum of 62 participants was required to maintain adequate power. Ultimately, 70 participants (35/group) were recruited to ensure robust statistical inference and to provide a buffer for attrition.

### Group Allocation and Masking

The PNO2PSP intervention required peer support groups with at least 3 participants. This requirement was primarily grounded in prior peer-support studies, which indicate that a minimum of 3 members is essential to create a collaborative atmosphere and foster a diverse exchange of knowledge and experiential sharing [31]. From a clinical feasibility perspective, this threshold also ensured that the support group remained functional and stable even if 1 member was temporarily unavailable or withdrew. Given these requirements, individual randomization was impractical for ensuring stable group formation among young- to middle-aged patients with breast cancer undergoing treatment. Consequently, a CRCT design was adopted, with weekly cohorts of surgical patients serving as natural clusters [41]. Participants scheduled for surgery in the following week were recruited every Sunday to form a weekly cohort. Random numbers were generated using R (R Core Team) in RStudio (Posit PBC). The allocation results for intervention and control groups were placed in sealed, opaque envelopes prepared in advance by an independent research assistant responsible for maintaining allocation concealment. After recruitment each week, the corresponding envelope was opened to determine group assignment. Since most patients were discharged by Friday of their surgery week, this cohort-based grouping minimized the risk of intervention contamination between the intervention and control groups. Due to the intervention’s nature, blinding of participants and providers was not feasible, and self-reported outcomes precluded blinding of outcome assessors. Thus, a single-blind design was used, with only statistical analysts blinded to group assignments to reduce bias.

### Interventions

The control group received routine care, which included standard discharge education and follow-up. The discharge education was primarily provided by nurses through verbal health education, covering topics such as postoperative wound care, protection of the affected limb, and rehabilitation exercises. Follow-up involved sending reminders to participants about regular dressing changes, encouraging adherence to chemotherapy schedules, and prompting them to attend follow-up visits.

The “Peer Support Intervention Manual” was distributed to the participants in the intervention group. Research assistants categorized participants and peer supporters into groups based on general demographic information, such as age, educational background, marital status, and reproductive history. Each group consisted of 2 peer supporters, 1 intervention nurse, and 3‐5 group members. The groups were then assigned to implement the intervention. The intervention was implemented according to the finalized version of the peer support intervention program developed in the earlier stages. The intervention details are shown in Multimedia Appendix 1.

### Evaluation

This study uses both quantitative and qualitative methods for outcome assessment. The quantitative evaluation primarily focuses on changes in the primary and secondary outcome measures through survey scales, while the qualitative evaluation involves interviews to understand participants’ experiences.

#### Quantitative Evaluation

##### Primary Outcome Measure

The primary outcome of this study was defined as the mean difference in the psychosocial adjustment total score at the 2-month follow-up (T2). This specific time point was prespecified because previous longitudinal studies have indicated that improvements in psychosocial outcomes are more robustly observable at this interval compared to baseline [23]. The Psychosocial Adjustment to Illness Scale (PAIS) was used to assess the level of psychosocial adjustment. This scale includes 46 items across 7 dimensions: health care orientation, vocational environment, domestic environment, sexual relationships, extended family relationships, social environment, and psychological distress. The total score ranges from 0 to 138, with higher scores indicating poorer psychosocial adjustment and a greater level of psychosocial problems. The Cronbach α coefficient of the scale is 0.86 [23].

##### Secondary Outcome Measures

The secondary outcomes included the individual dimension scores of psychosocial adjustment, as well as the scores for self-efficacy, social support, and various dimensions of coping modes across all follow-up time points. The corresponding measurement instruments for these variables are detailed below.

###### General Self-Efficacy Scale

The General Self-Efficacy Scale (GSES) measures the self-efficacy of young- to middle-aged patients with breast cancer. It consists of 10 items, rated using a Likert 4-point scale. The total score ranges from 10 to 40, with higher scores reflecting greater self-efficacy. The Cronbach α coefficient is 0.91 [42].

###### Social Support Rate Scale

The Social Support Rate Scale (SSRS) assesses social support levels, consisting of 10 items. Each item is rated on a 4-point Likert scale, with higher scores indicating higher social support. The Cronbach α coefficient for SSRS is 0.92 [43].

###### Medical Coping Modes Questionnaire

The Medical Coping Modes Questionnaire (MCMQ) evaluates coping strategies, with 20 items divided into 3 dimensions: confrontation, avoidance, and acceptance-resignation. Higher scores on each dimension indicate a stronger tendency to adopt the corresponding coping mode [44]. The Cronbach α coefficient for the Chinese version ranges from 0.68 to 0.89.

### Additional Data

In addition to the standardized scales, additional data were collected to provide information on the demographic and clinical characteristics of the participants and to assess treatment burden, as follows: (1) demographic information: sociodemographic data, including age, education level, marital status, residence, employment, and family income; (2) disease information survey: cancer type, disease stage, surgery type, treatment methods, lymph node involvement, and axillary lymph node dissection; and (3) treatment burden survey: patients’ treatment burden was assessed during the final follow-up (week 12) through a self-reported survey. This included treatment costs, unplanned hospital visits, outpatient visits, and total hospitalization time.

### Qualitative Evaluation

A purposeful sampling strategy was used to recruit participants from the intervention group. To ensure rich and diverse perspectives, participants of different ages, disease stages, and levels of psychosocial adjustment based on quantitative scores were invited. Semistructured, face-to-face, one-on-one interviews were conducted using an interview guide. Core questions included the following: (1) What is your impression of this project? (2) How has this project impacted your psychosocial adjustment? (3) What are the strengths and weaknesses of this project? The entire interview process was audio-recorded.

### Data Collection

Participants who were admitted to the hospital wards were selected according to the inclusion and exclusion criteria of this study. Once eligible patients were identified, the purpose and significance of the study were explained to them, informed consent was obtained, and a baseline data questionnaire was completed. The completed forms were checked for missing items on site, and once verified, they were numbered to ensure data quality. Eligible participants were randomly assigned to the intervention group or the control group, and interventions were conducted accordingly. Based on previous studies, this study established follow-up data collection at 3 time points: 4 weeks post surgery (T1), 8 weeks post surgery (T2), and 12 weeks post surgery (T3) [23]. At baseline, demographic information, disease information, psychosocial adjustment, self-efficacy, social support, and coping modes were collected. At T1, T2, and T3, psychosocial adjustment, self-efficacy, social support, and coping modes were reassessed. At T3, additional information on treatment costs, number of doctor visits, hospitalizations, and unplanned readmissions was collected.

For the qualitative part, participants were contacted 1‐2 days before the interview to explain the study’s purpose and procedures. Interviews were conducted after obtaining written informed consent. All interviews took place in a quiet, private room in the ward and were conducted individually by trained researchers with extensive qualitative interviewing experience. At the beginning of each interview, participants were informed that there were no right or wrong answers and were encouraged to share their experiences openly. They were also told that they could pause or stop the interview at any time if they felt uncomfortable. All data were anonymized to ensure confidentiality. The sample size for the qualitative phase was determined by data saturation. Data analysis was conducted alongside data collection, and recruitment ceased when no new themes emerged in 3 consecutive interviews [45].

### Data Analysis

Descriptive statistics were used to summarize participants’ baseline characteristics. Continuous variables were compared using independent samples *t* tests or Mann-Whitney *U* tests, while categorical variables were analyzed using Pearson chi-square (*χ*^2^) tests or Fisher exact tests. Baseline balance was assessed between the intervention and control groups, as well as between participants who completed the study and those lost to follow-up. This study followed the intention-to-treat principle, including all participants who were randomized. No formal imputation was performed for missing data. Generalized estimating equations (GEEs) use all available observations under the missing-at-random assumption and provide valid population-averaged estimates without requiring explicit imputation.

The GEE model was used to evaluate the intervention effect. “Week” was defined as the cluster variable to adjust for cohort-level correlations, and “TIME” was set as the repeated factor. Data were sorted by participants’ hospital identification numbers to ensure the integrity of the repeated measures structure in the model. TIME was treated as a categorical variable, and the group × time interaction effect was tested. The models specified a Gaussian identity link function with an exchangeable working correlation structure. To ensure the reliability of the inferences, robust (sandwich) SEs were used to provide unbiased parameter estimates. Clustering was explicitly modeled at the week level to account for potential correlation within cohorts. This approach ensures that the SEs are adjusted for the nested structure of the data.

The primary analysis consisted of unadjusted GEE models, which included group, time, and the group × time interaction as predictors. The control group and baseline (T0) served as the reference categories. To test the robustness of the primary findings against potential confounding—specifically the baseline imbalance observed in coping modes (avoidance)—sensitivity analyses were performed using adjusted GEE models. In these models, baseline coping modes (avoidance) were included as a covariate. These analyses confirmed that the statistical significance and direction of the intervention effects remained consistent across both models, reinforcing the reliability of the primary results. Further analysis revealed no significant interaction between group size and the intervention effect on the primary outcome. Statistical significance was defined as *P*<.05. All analyses were performed using R.

Qualitative data collection and analysis were conducted concurrently. All interviews were transcribed verbatim within 24 hours after completion. Data were analyzed using content analysis. Two researchers independently performed open coding, grouped similar codes into categories, and further abstracted them into core categories. Relationships among categories were examined during the abstraction process. Discrepancies were resolved through team discussion until consensus was reached, and triangulation was used to integrate qualitative and quantitative findings, allowing for a more comprehensive interpretation of how and why the intervention produced its observed effects [46].

### Quality Control

A standardized protocol for the implementation of the intervention was developed, and all team members involved in the intervention received uniform training to minimize inconsistencies during the execution of the plan. Research assistants participated in both online and offline group discussions throughout the intervention process. All offline interventions were video-recorded, online interventions were screen-recorded, and all voice chats were audio-recorded to ensure comprehensive documentation. The research team held meetings at least once a week to identify issues and difficulties in the implementation of the research protocol. All peer supporters underwent uniform training and assessment. Additionally, intervention guidelines were provided to assist them in the implementation process.

To minimize contamination between groups, a cluster enrollment strategy based on admission week was adopted. Patients in the intervention and control groups were recruited in different weeks, and due to the fixed surgical admission and discharge schedule, participants from different weeks did not overlap during hospitalization. This temporal separation reduced opportunities for cross-group interaction. In addition, all participants signed a confidentiality agreement before the intervention, which emphasized that all information about intervention content and personal experiences of group members should not be disclosed to individuals outside the group. Intervention delivery was restricted to designated facilitators, and no crossover of intervention staff occurred between study arms.

### Ethical Considerations

This study complied with the principles of the Declaration of Helsinki and relevant Chinese regulations governing clinical research. It was approved by the Ethics Committee of the School of Nursing, Sun Yat-sen University, Guangzhou, China (approval no L2023SYSU-HL-019) and registered with the China Clinical Trial Registry. All relevant departments in the hospital approved the study. Participants in the study signed a paper copy of the informed consent before enrollment. They were provided with detailed information about the study’s objectives, process, potential risks and benefits, their right to withdraw at any time, and data collection methods. The data obtained in the study were handled in accordance with confidentiality principles. All data were anonymized before analysis. Personal and clinical information was securely stored, and access was restricted to authorized members of the research team only. Identifiable features or images of patients are not included in the paper and supplementary materials. Participants did not receive any financial compensation for their involvement in the study. However, they were provided with free educational support as part of the research process. To ensure that all participants benefit equitably, after the data collection was completed, the participants in the control group received the intervention manual and were added to online peer support groups.

## Results

### Enrollment

During the study, 90 young- and middle-aged patients with breast cancer who met the inclusion criteria were approached. Among them, 20 refused to participate, 10 expressed no interest, 7 were unwilling to complete the questionnaires, and 3 did not have time to participate in the intervention. As a result, a total of 70 participants from 6 recruitment clusters (weeks) were included in the final analysis. For the primary outcome, the observed intraclass correlation was 0.0303, which is below the threshold of 0.05 and therefore indicates a small degree of clustering according to previous studies [47]. Given an average cluster size of 11.67, the DE was calculated to be 1.323. Post hoc power analysis using PASS (NCSS LLC) demonstrated that with this DE and sample size, the study achieved a statistical power of 0.875 to detect the intervention effect on the primary outcome, confirming the adequacy of the sample size for robust inference.

### Follow-Up and Attrition

**Figure 1.**
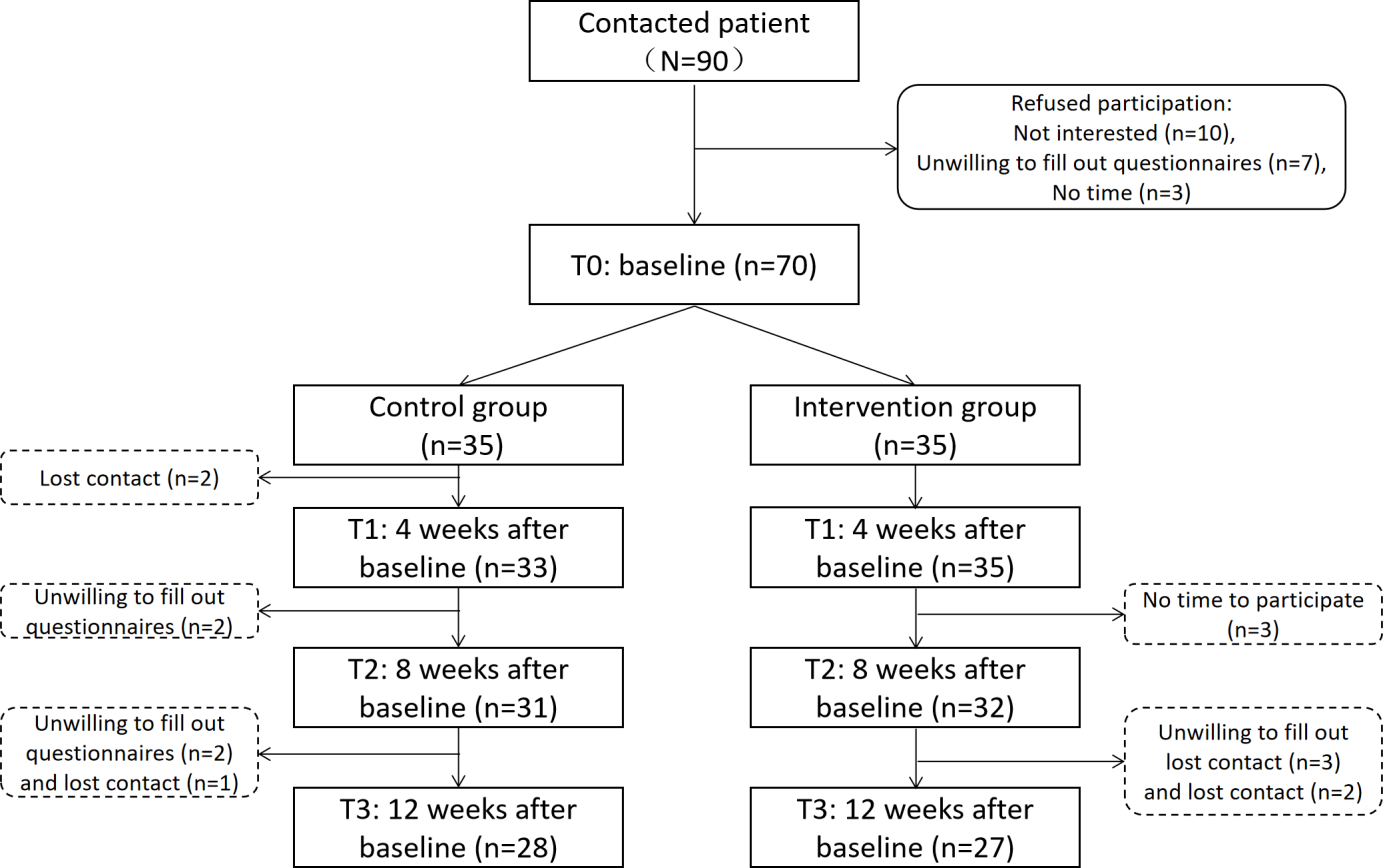
CONSORT flow diagram. Enrollment and follow-up situations of patients in the intervention group and the control group.

At T1, 33 control group participants and all 35 intervention group participants completed the follow-up, with 2 losses in the control group. At T2, 31 control and 32 intervention participants completed the follow-up, with 4 and 3 losses, respectively. At T3, 28 control and 27 intervention participants completed the follow-up, with 7 and 8 losses, respectively. Overall, the attrition rate for the control group was calculated as 12.4%. The main reasons for attrition were loss of contact or unwillingness to complete the follow-up questionnaire. The attrition rate for the intervention group was 10.5%. The main reasons for attrition were unwillingness to continue participation, lack of time to complete the questionnaire, or loss of contact. A flowchart of patient recruitment, enrollment, follow-up, and attrition is shown in Figure 1.

### Baseline Data Balance Test

The baseline data comparison between the follow-up completion group and the lost-to-follow-up group showed no statistically significant differences in general information, disease, and treatment data (*P*>.05; shown in Table 1). However, there was a statistically significant difference in social support (*P=.*038). The comparison of baseline data between the intervention group and the control group revealed no statistically significant differences in general information, disease, and treatment data (*P*>.05). However, there was a statistically significant difference in the avoidance dimension of coping strategies (*P=.*003; shown in Table 2). Subsequently, the GEE analysis was conducted, with these scores used as a covariate to adjust the results (shown in Table 3).

**Table 1.** Balance test of baseline data between the follow-up completion group and the lost-to-follow-up group (N=70).

Project and group	Lost-to-follow-up group (n=16)	Follow-up completion group (n=54)	Statistics	*P* value
Age (years), mean (SD)	43.38 (7.974)	44.07 (7.427)	–0.33 (68)^a^	.76
Number of children, n (%)			0.44 (2)^b^	.80
0	2 (12.5)	4 (7.4)		
1	6 (37.5)	20 (37)		
≥2	8 (50)	30 (55.6)		
Marital status, n (%)			0.07 (1)^c^	.79
Married	16 (100)	51 (94.4)		
Unmarried	0 (0)	3 (5.6)		
Educational attainment, n (%)			0.75 (2)^b^	.69
Junior high school or below	4 (25)	9 (16.7)		
High school or technical secondary	5 (31.2)	22 (40.7)		
College or above	7 (43.8)	23 (42.6)		
Occupation type, n (%)			1.26 (4)^b^	.87
Enterprise employee	4 (25)	14 (25.9)		
Farmer or worker	3 (18.8)	5 (9.3)		
Government employee	4 (25)	14 (25.9)		
Freelancer	3 (18.8)	11 (20.4)		
Unemployed	2 (12.4)	10 (18.5)		
Place of residence, n (%)			0.16 (1)^b^	.69
Town	12 (75)	43 (79.6)		
Rural area	4 (25)	11 (20.4)		
Religious belief, n (%)			0.51 (1)^c^	.48
None	16 (100)	49 (90.7)		
Yes	0 (0)	5 (9.3)		
Monthly household income per capita (CNY), n (%)			3.62 (3)^b^	.31
<3000 (US $420)	5 (31.3)	11 20.3)		
3000-5000 (US $420-700)	1 (6.3)	15 (27.8)		
5001-10,000 (US $700-1400)	6 (37.4)	19 (35.2)		
＞10,000 (US $1400)	4 (25)	9 (16.7)		
Tumor grade, n (%)			0.51 (2)^b^	.77
I	2 (12.5)	11 (20.4)		
II	8 (50)	24 (44.4)		
III	6 (37.5)	19 (35.2)		
Lymph node metastasis, n (%)			0.81 (2)^b^	.37
No	6 (37.5)	14 (25.9)		
Yes	10 (62.5)	40 (74.1)		
Axillary lymph node dissection, n (%)			0.63 (1)^c^	.43
Yes	1 (6.3)	10 (18.5)		
No	15 (93.7)	44 (81.5)		
Triple-negative, n (%)			—^e^	1^d^
Yes	1 (6.3)	4 (7.4)		
No	15 (93.7)	50 (92.6)		
Lesion location, n (%)			—	.34^d^
Left only	11 (68.8)	29 (53.7)		
Right only	4 (25)	23 (42.6)		
Bilateral	1 (6.2)	2 (3.7)		
Type of surgery, n (%)			—	.09^d^
Breast-conserving surgery	9 (56.3)	36 (66.7)		
Modified radical surgery	6 (37.4)	7 (13)		
Total resection	1 (6.3)	11 (20.3)		
Received chemotherapy, n (%)			0.10 (1)^b^	.76
Yes	10 (62.5)	36 (66.7)		
No	6 (37.5)	18 (33.3)		
Self-efficacy, mean (SD)	25.88 (7.35)	26.76 (6.87)	–0.45 (68)^a^	.66
Social support, mean (SD)	40.94 (7.63)	45.19 (6.88)	–2.12 (68)^a^	.04
Coping strategies, n (%)				
Facing	20.13 (3.76)	20.22 (2.93)	–0.11 (68)^a^	.91
Avoidance	17.69 (2.70)	17.35 (2.74)	0.43 (68)^a^	.67
Yield	8.75 (3.30)	7.98 (2.33)	1.05 (68)^a^	.30
Psychosocial adjustment, mean (SD)	49.56 (20.32)	43.94 (17.06)	1.11 (68)^a^	.27

a *t* test.

b Pearson chi-square test.

c Continuous correction chi-square test: the expected value of the cell is <5.

d Fisher exact probability method: the expected value of the cell is <1.

e Not available.

**Table 2.** Balance test of baseline data between the intervention group and the control group (N=70).

Project and group	Intervention group (n=35)	Control group (n=35)	Statistics	*P* value
Age (years), mean (SD)	42.83 (6.719)	45.00 (8.164)	–1.21 (68)^a^	.23
Number of children, n (%)			0.26 (2)^c^	.88
0	3 (8.6)	3 (8.6)		
1	14 (40.0)	12 (34.3)		
≥2	18 (51.4)	20 (57.1)		
Marital status, n (%)			0 (1)^c^	≥.99
Married	33 (94.3)	34 (97.1)		
Unmarried	2 (5.7)	1 (2.9)		
Educational attainment, n (%)			0.54 (2)^b^	.76
Junior high school or below	7 (20)	6 (17.1)		
High school or technical secondary	12 (34.3)	15 (42.9)		
College or above	16 (45.7)	14 (40)		
Occupation type, n (%)			2.87 (4)^c^	.58
Enterprise employee	11 (31.4)	7 (20)		
Farmer or worker	5 (14.3)	3 (8.6)		
Government employee	9 (25.7)	9 (25.7)		
Freelancer	5 (14.3)	9 (25.7)		
Unemployed	5 (14.3)	7 (20)		
Place of residence, n (%)			0.09 (1)^b^	.77
Town	27 (77.1)	28 (80)		
Rural area	8 (22.9)	7 (20)		
Religious belief, n (%)			0.22 (1)^c^	.64
None	32 (91.4)	33 (94.3)		
Yes	3 (8.6)	2 (5.7)		
Monthly household income per capita (CNY)^e^, n (%)			2.96 (3)^b^	.40
＜3000 (US $420)	8 (22.9)	8 (22.9)		
3000-5000 (US $420-US $700)	6 (17.1)	10 (28.6)		
5001-10,000 (US $700-US $1400)	12 (34.3)	13 (37.1)		
＞10,000 (US $1400)	9 (25.7)	4 (11.4)		
Tumor grade, n (%)			2.18 (2)^b^	.34
I	8 (22.9)	5 (14.3)		
II	13 (37.1)	19 (54.3)		
III	14 (40)	11 (31.4)		
Axillary lymph node dissection, n (%)			0.97 (1)^b^	.32
Yes	7 (20)	4 (11.4)		
No	28 (80)	31 (88.6)		
Triple-negative, n (%)			0 (1)^c^	>.99
Yes	3 (8.6)	2 (5.7)		
No	32 (91.4)	33 (94.3)		
Lesion location, n (%)			3.06 (2)^d^	.25
Left only	18 (51.4)	22 (62.9)		
Right only	14 (40)	13 (37.1)		
Bilateral	3 (8.6)	0 (0)		
Type of surgery, n (%)			1.58 (2)^b^	.45
Breast-conserving surgery	25 (71.4)	20 (57.1)		
Modified radical surgery	5 (14.3)	8 (22.9)		
Total resection	5 (14.3)	7 (20)		
Received chemotherapy, n (%)			0.25 (1)^b^	.62
Yes	24 (68.6)	22 (62.9)		
No	11 (31.4)	13 (37.1)		
Self-efficacy, mean (SD)	25.94 (6.86)	27.17 (7.06)	–0.74 (68)^a^	.46
Social support, mean (SD)	43.77 (7.38)	44.66 (7.15)	–0.51 (68)^a^	.61
Coping strategies, mean (SD)				
Facing	20.20 (2.77)	20.20 (3.45)	0 (68)^a^	≥.99
Avoidance	16.49 (2.36)	18.37 (2.76)	–3.08 (68)^a^	.003
Yield	8.23 (2.28)	8.09 (2.87)	0.23 (68)^a^	.82
Psychosocial adjustment, mean (SD)				
Total score	46.60 (17.14)	43.86 (18.69)	0.64 (68)^a^	.53
Health care	9.03 (3.20)	8.86 (2.93)	0.23 (68)^a^	.82
Occupational environment	7.11 (3.11)	6.00 (3.19)	1.48 (68)^a^	.14
Family environment	6.03 (3.22)	5.09 (2.97)	1.27 (68)^a^	.21
Sexual relationship	6.00 (2.77)	6.66 (3.46)	–0.88 (68)^a^	.38
Expand family relationships	2.77 (2.37)	2.11 (1.92)	1.27 (68)^a^	.21
Social relationship	7.46 (4.58)	6.57 (5.26)	0.75 (68)^a^	.46
Psychological distress	8.20 (4.68)	8.57 (4.86)	–0.33 (68)^a^	.75

a *t* test.

b Pearson chi-square test.

c Continuous correction chi-square test: the expected value of the cell is <5.

d Fisher exact probability method: the expected value of the cell is <1.

**Table 3.** Results of generalized estimating equation models for psychosocial adjustment and scores of psychosocial adjustment and secondary outcomes in 2 groups of young- and middle-aged patients with breast cancer (N=70).

Variable and time	Control group (n=35)	Intervention group (n=35)	Crude model						Adjusted model					
			Group effect		Time effect		Interaction effect		Group effect		Time effect		Interaction effect	
			Wald *χ²* (*df*)	*P *value	Wald *χ²* (*df*)	*P* value	Wald *χ²* (*df*)	*P* value	Wald *χ²* (*df*)	*P* value	Wald *χ²* (*df*)	*P* value	Wald *χ²* (*df*)	*P *value
Psychosocial adjustment, mean (SD)			6.83 (1)	.009	41.03 (3)	<.001	19.03 (3)	<.001	3.62 (1)	.06	48.15 (3)	<.001	18.39 (3)	<.001
T0	46.60 (17.14)	43.86 (18.69)												
T1	52.91 (18.65)	48.03 (18.13)												
T2	55.32 (16.36)	43.31 (14.67)												
T3	52.64 (17.47)	42.44 (17.55)												
Health orientation, mean (SD)			8.41 (1)	.004	3.82 (3)	.28	477.87 (3)	<.001	7.09 (1)	.008	4.73 (3)	.19	248.08 (3)	<.001
T0	9.03 (3.20)	8.86 (2.93)												
T1	9.82 (2.89)	8.40 (2.66)												
T2	9.65 (2.46)	9.13 (2.85)												
T3	9.14 (2.77)	9.11 (2.97)												
Vocational environment, mean (SD)			7.47 (1)	.006	23.91 (3)	0	5.60 (3)	.13	4.49 (1)	.03	19.68 (3)	<.001	5.43 (3)	.14
T0	7.11 (3.11)	6.00 (3.19)												
T1	8.73 (3.57)	7.43 (3.90)												
T2	8.81 (2.88)	6.41 (3.40)												
T3	7.68 (3.43)	6.07 (3.74)												
Domestic environment, mean (SD)			5.40 (1)	.02	10.38 (3)	.02	24.30 (3)	<.001	2.01 (1)	.16	10.42 (3)	.02	29.67 (3)	<.001
T0	6.03 (3.22)	5.09 (2.97)												
T1	6.12 (3.51)	6.49 (3.53)												
T2	7.19 (3.98)	5.44 (2.77)												
T3	7.07 (3.85)	5.07 (3.41)												
Sexual relationship, mean (SD)			0.48 (1)	.49	67.19 (3)	<.001	12.36 (3)	.006	0.21 (1)	.65	64.38 (3)	<.001	12.140 (3)	.007
T0	6.00 (2.77)	6.66 (3.47)												
T1	7.64 (3.44)	8.00 (3.24)												
T2	8.65 (3.23)	6.97 (2.95)												
T3	8.11 (3.06)	6.74 (3.10)												
Expand family relationships, mean (SD)			10.63 (1)	.001	29.16 (3)	<.001	8.99 (3)	.03	8.06 (1)	.005	29.48 (3)	<.001	7.50 (3)	.06
T0	2.77 (2.38)	2.11 (1.92)												
T1	3.52 (2.35)	2.49 (1.96)												
T2	3.42 (2.69)	2.31 (1.87)												
T3	3.96 (2.66)	2.48 (1.99)												
Social environment, mean (SD)			5.24 (1)	.02	3.28 (3)	.35	11.99 (3)	.007	1.82 (1)	.18	3.61 (3)	.31	11.50 (3)	.009
T0	7.46 (4.58)	6.57 (5.26)												
T1	8.21 (3.88)	7.40 (4.43)												
T2	8.81 (3.19)	6.69 (4.19)												
T3	8.57 (3.86)	6.78 (4.49)												
Psychological distress, mean (SD)			1.43 (1)	.23	10.65 (3)	.01	41.20 (3)	<.001	0.87 (1)	.35	10.99 (3)	.01	40.41 (3)	<.001
T0	8.20 (4.68)	8.57 (4.86)												
T1	8.88 (5.15)	7.83 (4.88)												
T2	8.81 (4.21)	6.38 (4.08)												
T3	8.11 (4.45)	6.19 (4.45)												
Self-efficacy, mean (SD)			2.57 (1)	.11	10.93 (3)	.01	2.42 (3)	.49	0.05 (1)	.83	12.35 (3)	.006	2.86 (3)	.42
T0	25.94 (6.87)	27.17 (7.06)												
T1	27.00 (6.72)	27.23 (6.66)												
T2	24.84 (7.19)	26.47 (6.68)												
T3	25.14 (7.24)	26.41 (6.58)												
Social support, mean (SD)			2.84 (1)	.09	3.95 (3)	.27	94.25 (3)	<.001	5.11 (1)	.02	3.59 (3)	.31	101.90 (3)	<.001
T0	43.77 (7.38)	44.66 (7.15)												
T1	43.55 (8.05)	45.40 (7.73)												
T2	41.48 (7.82)	45.81 (6.68)												
T3	43.46 (7.39)	46.00 (7.12)												
Coping mode to confrontation, mean (SD)			3.44 (1)	.06	1.34 (3)	.72	29.77 (3)	<.001	2.47 (1)	.12	1.64 (3)	.65	28.29 (3)	<.001
T0	20.20 (2.77)	20.20 (3.45)												
T1	20.36 (2.86)	20.66 (3.13)												
T2	19.87 (3.30)	21.34 (3.42)												
T3	19.68 (3.07)	21.37 (3.59)												
Coping mode to avoidance, mean (SD)			2.05 (1)	.15	85.93 (3)	<.001	18.72 (3)	<.001	2.05 (1)	.15	85.93 (3)	0	18.72 (3)	<.001
T0	16.49 (2.36)	18.37 (2.76)												
T1	17.88 (2.03)	18.06 (2.59)												
T2	17.26 (3.04)	16.91 (2.74)												
T3	17.32 (2.18)	17.41 (2.90)												
Coping mode to acceptance to resignation, mean (SD)			2.94 (1)	.09	5.417 (3)	.14	17.55 (3)	.001	1.37 (1)	.24	6.68 (3)	.08	12.46 (3)	.006
T0	8.23 (2.28)	8.09 (2.87)												
T1	8.33 (2.87)	7.66 (2.61)												
T2	8.55 (2.53)	7.63 (2.41)												
T3	8.86 (3.10)	7.63 (3.13)												

### Quantitative Outcomes

The results of the GEE model, intergroup comparison analysis, and within-group comparison analysis in this study are shown in Table 3.

### Psychosocial Adjustment

The intervention group had significantly lower psychosocial adjustment scores than the control group at T2 (*P*<.001) and T3 (*P*=.006).

### Secondary Outcome

**Figure 2.**
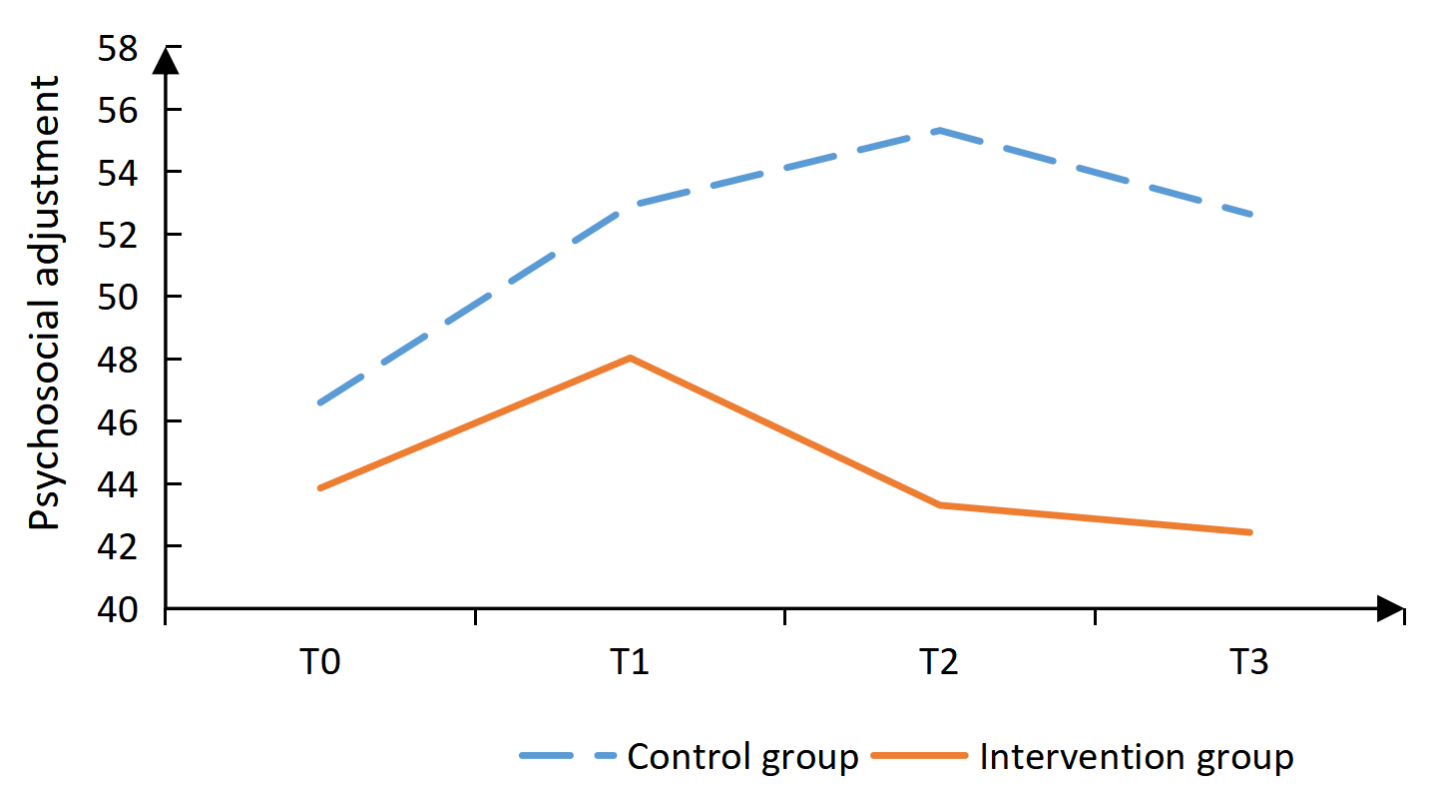
Results of the intervention effect evaluation: total scores.

**Figure 3.**
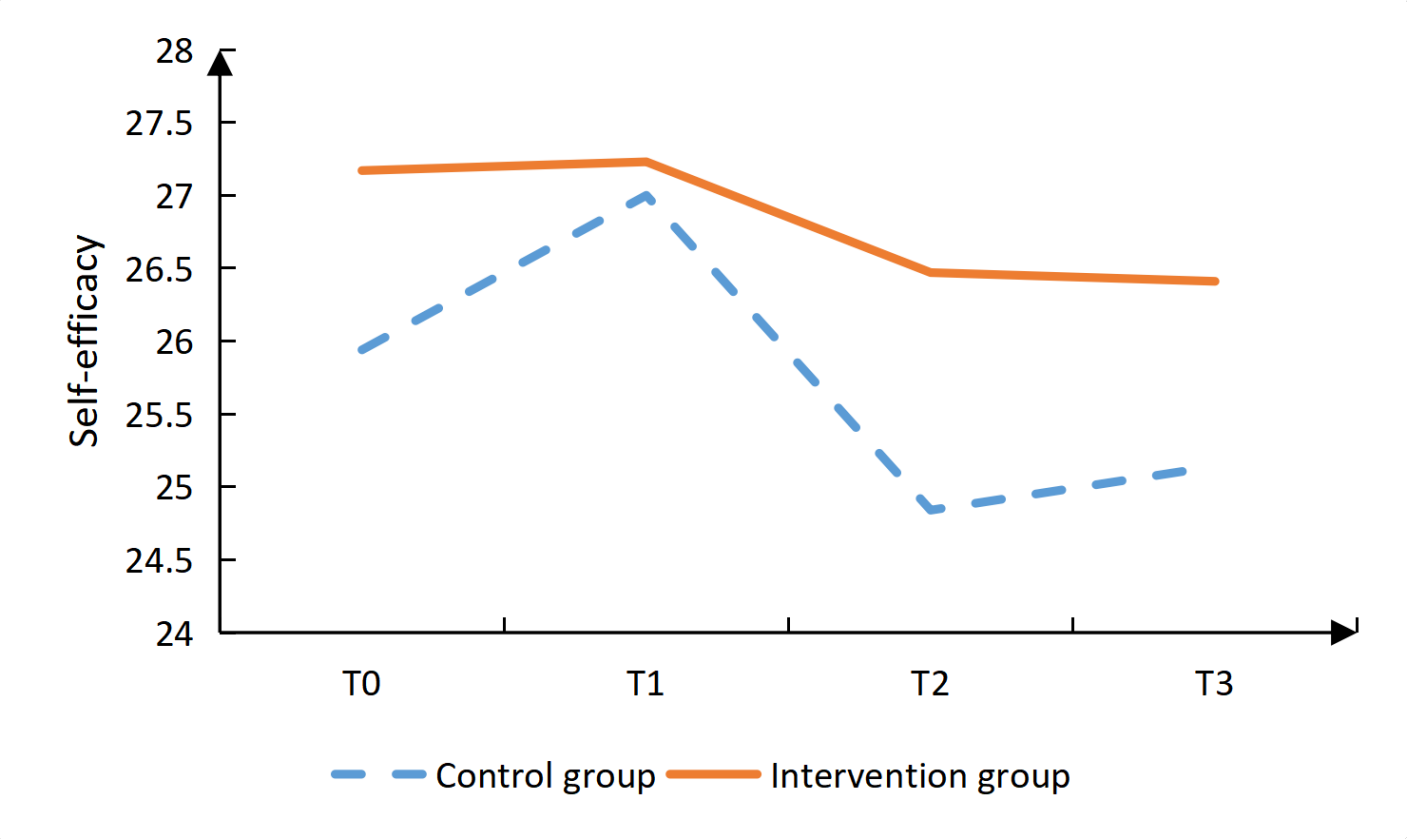
Results of the intervention effect evaluation: self-efficacy.

**Figure 4.**
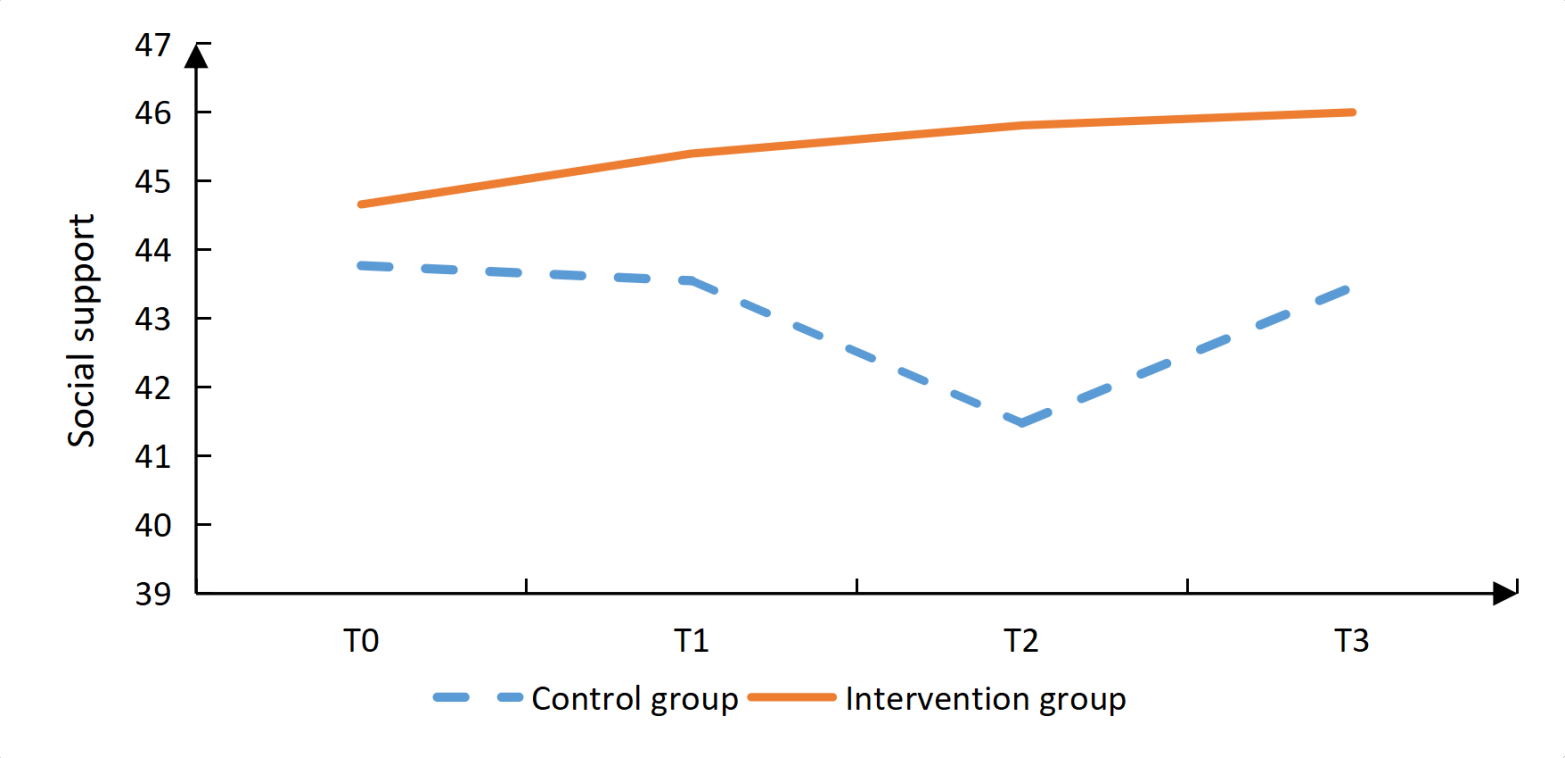
Results of the intervention effect evaluation: social support.

**Figure 5.**
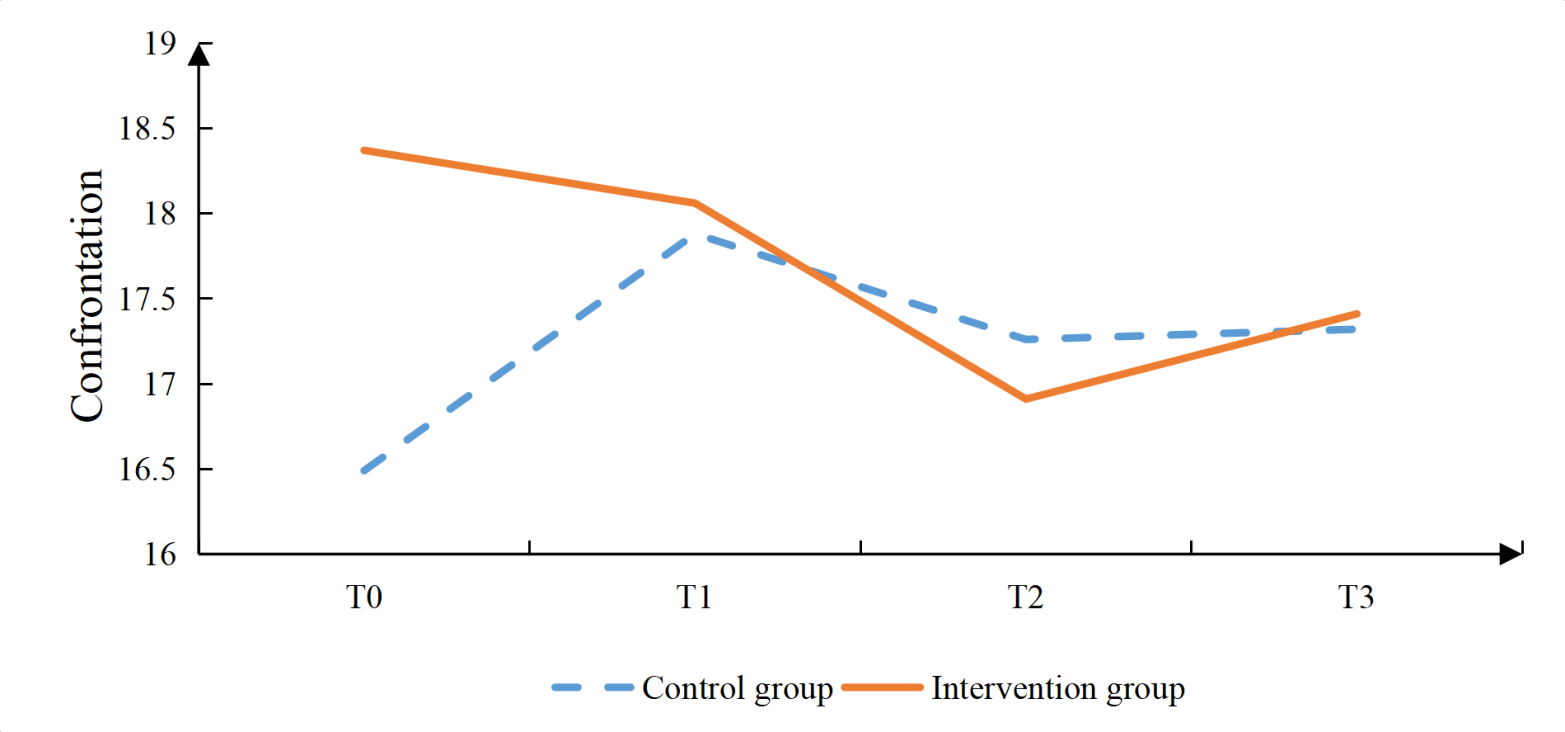
Results of the intervention effect evaluation: confrontation.

**Figure 6.**
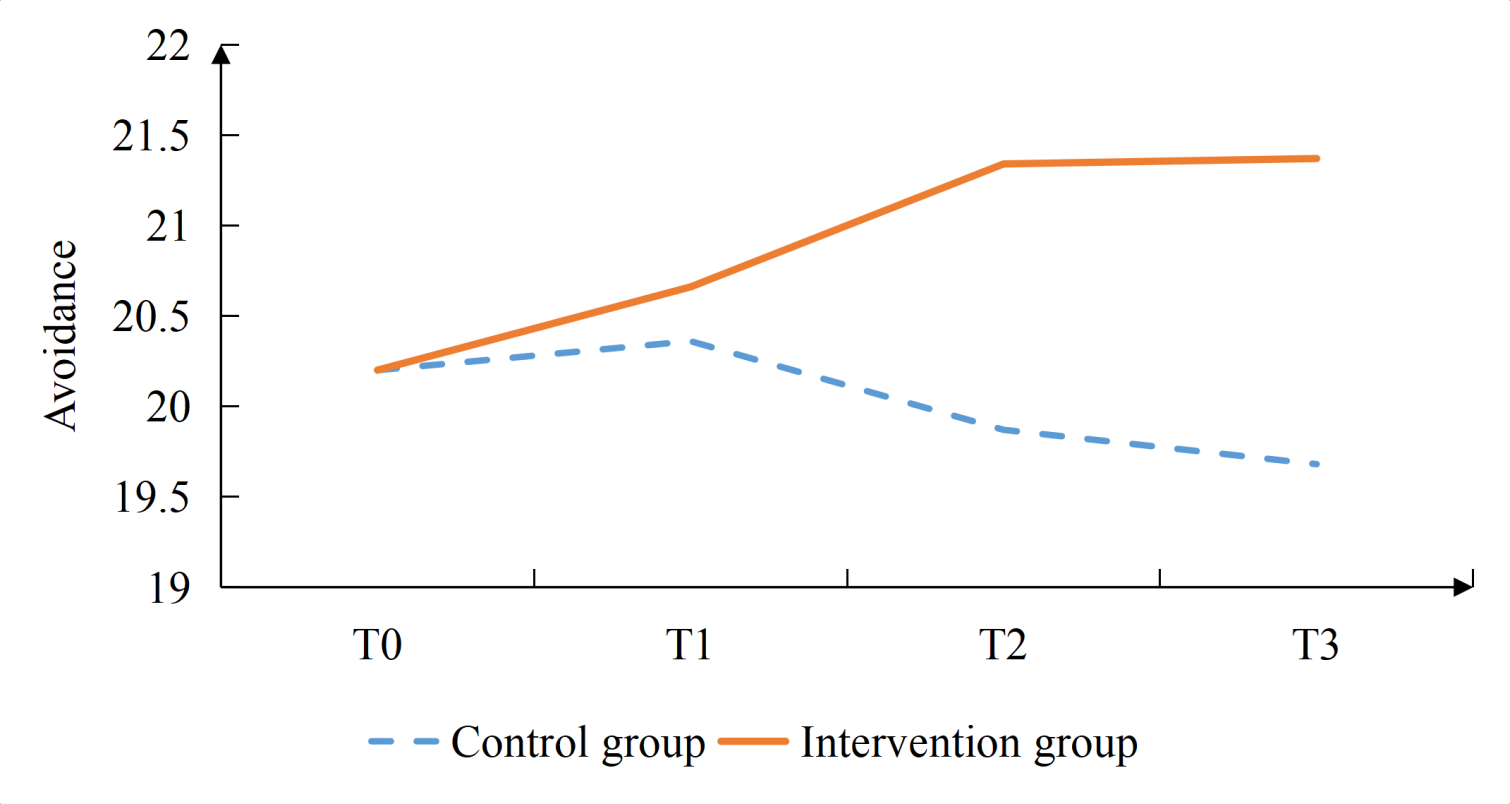
Results of the intervention effect evaluation: avoidance.

**Figure 7.**
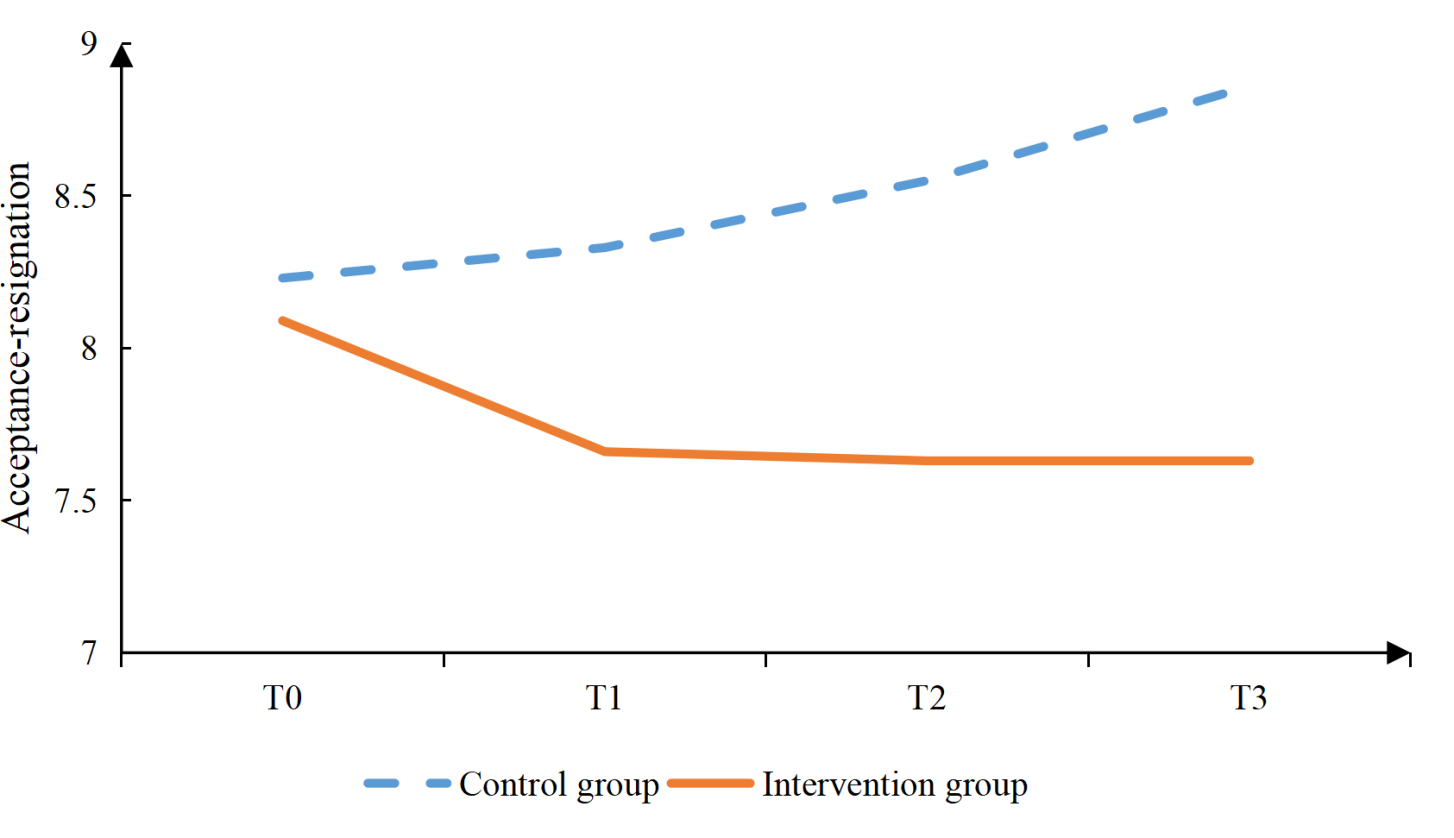
Results of the intervention effect evaluation: acceptance-resignation.

Additionally, the intervention group had significantly lower scores in the vocational environment dimension at T3 (*P*=.020), in the sexual relationship dimension at T2 (*P*=.001) and T3 (*P*=.002), as well as in the psychological distress dimension at T2 (*P*<.001) and T3 (*P*<.001; Table 4). The intervention group reported significantly higher social support scores than the control group (*P*=.006) at T2. Confrontation coping scores were also significantly higher in the intervention group (*P*=.019) at T2, (*P*=.002) at T3. In contrast, avoidance coping scores were significantly lower in the intervention group (*P*=.004, .006, and .017) at T1, T2, and T3, respectively. No significant differences were observed between groups in self-efficacy or acceptance-resignation coping at any time point (Table 4, Figures 2-7, and Multimedia Appendix 2).

**Table 4.** Comparison of generalized estimating equations for psychosocial adjustment and secondary outcomes between groups at different time points.

Outcome	Crude model			Adjusted model		
	B (95% CI)	Wald *χ²* (*df*)	*P* value	B (95% CI)	Wald *χ²* (*df*)	*P* value
Psychosocial adjustment						
Group	–2.705 (–8.366 to 2.956)	0.88 (1)	.35	–1.558 (–8.173 to 5.056)	0.21 (1)	.64
Time1	6.315 (2.949 to 9.681)	13.52 (1)	<.001	6.277 (2.872 to 9.683)	13.052 (1)	<.001
Time2	8.829 (4.533 to 13.124)	16.23 (1)	<.001	8.834 (4.487 to 13.180)	15.87 (1)	<.001
Time3	6.09 (1.329 to 10.850)	6.29 (1)	.01	6.117 (1.280 to 10.953)	6.15 (1)	.01
Group×Time1	–2.143 (–5.936 to 1.649)	1.23 (1)	.27	–2.106 (–5.894 to 1.683)	1.19 (1)	.28
Group×Time2	–9.406 (–14.172 to –4.640)	14.97 (1)	<.001	–9.582 (–14.405 to –4.759)	15.16 (1)	<.001
Group×Time3	–7.571 (–12.993 to –2.150)	7.49 (1)	.006	–7.539 (–12.995 to –2.082)	7.33 (1)	.007
Health care						
Group	–0.151 (–0.952 to 0.651)	0.14 (1)	.71	–0.190 (–1.156 to 0.775)	0.15 (1)	.70
Time1	0.75 (–0.571 to 2.071)	1.24 (1)	.27	0.753 (–0.566 to 2.072)	1.25 (1)	.26
Time2	0.611 (–0.640 to 1.863)	0.92 (1)	.34	0.615 (–0.623 to 1.853)	0.95 (1)	.33
Time3	0.094 (–1.512 to 1.700)	0.01 (1)	.91	0.096 (–1.508 to 1.699)	0.01 (1)	.91
Group×Time1	–1.207 (–2.528 to 0.115)	3.20 (1)	.07	–1.210 (–2.530 to 0.110)	3.23 (1)	.07
Group×Time2	–0.369 (–1.975 to 1.236)	0.20 (1)	.65	–0.364 (–1.978 to 1.250)	0.20 (1)	.66
Group×Time3	0.152 (–1.696 to 2.001)	0.03 (1)	.87	0.146 (–1.689 to 1.982)	0.02 (1)	.88
Occupational environment						
Group	–1.109 (–2.099 to –0.118)	4.81 (1)	.03	–0.944 (–1.908 to 0.021)	3.68 (1)	.06
Time1	1.625 (0.351 to 2.899)	6.25 (1)	.01	1.621 (0.348 to 2.894)	6.23 (1)	.01
Time2	1.715 (1.400 to 2.030)	113.74 (1)	<.001	1.715 (1.409 to 2.020)	121.06 (1)	<.001
Time3	0.579 (0.274 to 0.884)	13.86 (1)	<.001	0.583 (0.266 to 0.900)	13.02 (1)	<.001
Group×Time1	–0.196 (–2.035 to 1.642)	0.04 (1)	.83	–0.192 (–2.036 to 1.652)	0.04 (1)	.84
Group×Time2	–1.302 (–2.952 to 0.347)	2.40 (1)	.12	–1.324 (–2.942 to 0.293)	2.57 (1)	.11
Group×Time3	–0.521 (–0.960 to ‐0.081)	5.40 (1)	.02	–0.515 (–0.970 to –0.060)	4.92 (1)	.03
Domestic environment						
Group	–0.941 (–1.726 to –0.157)	5.54 (1)	.02	–0.596 (–1.427 to 0.236)	1.97 (1)	.16
Time1	0.091 (–0.242 to 0.424)	0.29 (1)	.59	0.077 (–0.286 to 0.440)	0.17 (1)	.68
Time2	1.167 (–0.014 to 2.348)	3.75 (1)	.05	1.161 (–0.029 to 2.351)	3.66 (1)	.06
Time3	1.043 (–0.182 to 2.268)	2.79 (1)	.10	1.047 (–0.189 to 2.282)	2.76 (1)	.10
Group×Time1	1.309 (0.619 to 1.998)	13.84 (1)	<.001	1.323 (0.633 to 2.013)	14.11 (1)	<.001
Group×Time2	–0.82 (–2.444 to 0.804)	0.98 (1)	.32	–0.872 (–2.509 to 0.766)	1.09 (1)	.30
Group×Time3	–1.056 (–2.496 to 0.384)	2.07 (1)	.15	–1.038 (–2.496 to 0.420)	1.95 (1)	.16
Sexual relationship						
Group	0.672 (–0.842 to 2.187)	0.76 (1)	.38	0.764 (–0.977 to 2.505)	0.74 (1)	.39
Time1	1.635 (0.932 to 2.339)	20.75 (1)	<.001	1.632 (0.933 to 2.330)	20.96 (1)	<.001
Time2	2.661 (2.176 to 3.145)	116.01 (1)	<.001	2.66 (2.171 to 3.149)	113.627 (1)	<.001
Time3	2.113 (1.241 to 2.985)	22.57 (1)	<.001	2.115 (1.234 to 2.996)	22.129 (1)	<.001
Group×Time1	–0.292 (–1.109 to 0.524)	0.49 (1)	.48	–0.289 (–1.099 to 0.521)	0.489 (1)	.49
Group×Time2	–2.322 (–3.634 to –1.009)	12.03 (1)	.001	–2.332 (–3.660 to –1.004)	11.849 (1)	.001
Group×Time3	–2.048 (–3.318 to –0.779)	9.99 (1)	.002	–2.045 (–3.314 to –0.775)	9.969 (1)	.002
Expand family relationships						
Group	–0.659 (–1.298 to –0.020)	4.09 (1)	.04	–0.635 (–0.950 to –0.320)	15.63 (1)	<.001
Time1	0.744 (0.108 to 1.379)	5.26 (1)	.02	0.743 (0.098 to 1.388)	5.10 (1)	.02
Time2	0.664 (–1.141 to 2.470)	0.52 (1)	.47	0.665 (–1.146 to 2.476)	0.52 (1)	.47
Time3	1.2 (–0.277 to 2.677)	2.54 (1)	.11	1.201 (–0.276 to 2.677)	2.54 (1)	.11
Group×Time1	–0.372 (–1.061 to 0.316)	1.12 (1)	.29	–0.372 (–1.070 to 0.327)	1.09 (1)	.30
Group×Time2	–0.47 (–2.293 to 1.353)	0.26 (1)	.61	–0.474 (–2.274 to 1.325)	0.27 (1)	.61
Group×Time3	–0.835 (–2.393 to 0.722)	1.10 (1)	.29	–0.834 (–2.405 to 0.736)	1.08 (1)	.30
Social environment						
Group	–0.88 (–3.076 to 1.316)	0.62 (1)	.43	–0.458 (–2.776 to 1.860)	0.15 (1)	.70
Time1	0.758 (–0.654 to 2.170)	1.11 (1)	.29	0.746 (–0.688 to 2.180)	1.04 (1)	.31
Time2	1.354 (–0.177 to 2.886)	3.01 (1)	.08	1.349 (–0.180 to 2.877)	2.99 (1)	.08
Time3	1.118 (0.803 to 1.433)	48.37 (1)	<.001	1.125 (0.841 to 1.410)	60.21 (1)	<.001
Group×Time1	0.071 (–1.889 to 2.030)	0.01 (1)	.94	0.083 (–1.890 to 2.055)	0.01 (1)	.93
Group×Time2	–1.245 (–3.153 to 0.664)	1.63 (1)	.20	–1.304 (–3.200 to 0.592)	1.82 (1)	.18
Group×Time3	–0.919 (–2.248 to 0.409)	1.84 (1)	.18	–0.905 (–2.216 to 0.407)	1.83 (1)	.18
Psychological distress						
Group	0.393 (–1.581 to 2.367)	0.15 (1)	.70	0.608 (–1.665 to 2.881)	0.28 (1)	.60
Time1	0.665 (–0.991 to 2.321)	0.62 (1)	.43	0.657 (–0.993 to 2.306)	0.61 (1)	.44
Time2	0.661 (0.217 to 1.106)	8.51 (1)	.004	0.660 (0.234 to 1.086)	9.22 (1)	.002
Time3	–0.076 (–0.566 to 0.414)	0.09 (1)	.76	–0.072 (–0.564 to 0.419)	0.08 (1)	.77
Group×Time1	–1.408 (–3.339 to 0.524)	2.04 (1)	.15	–1.400 (–3.313 to 0.514)	2.06 (1)	.15
Group×Time2	–2.904 (–4.089 to –1.718)	23.05 (1)	<.001	–2.934 (–4.185 to –1.684)	21.15 (1)	<.001
Group×Time3	–2.343 (–3.112 to –1.574)	35.68 (1)	<.001	–2.334 (–3.106 to –1.562)	35.10 (1)	<.001
Self-efficacy						
Group	–0.88 (–3.076 to 1.316)	0.62 (1)	.43	0.344 (–1.016 to 1.703)	0.25 (1)	.62
Time1	0.758 (–0.654 to 2.170)	1.11 (1)	.29	1.091 (–2.291 to 4.472)	0.40 (1)	.58
Time2	1.354 (–0.177 to 2.886)	3.01 (1)	.08	–1.008 (–3.498 to 1.481)	0.63 (1)	.43
Time3	1.118 (0.803 to 1.433)	48.37 (1)	<.001	–0.777 (–3.003 to 1.450)	0.47 (1)	.49
Group×Time1	0.071 (–1.889 to 2.030)	0.01 (1)	.94	–1.033 (–4.473 to 2.406)	0.35 (1)	.56
Group×Time2	–1.245 (–3.153 to 0.664)	1.63 (1)	.20	0.443 (–2.553 to 3.440)	0.08 (1)	.77
Group×Time3	–0.919 (–2.248 to 0.409)	1.84 (1)	.18	–0.049 (–2.430 to 2.333)	0.002 (1)	.97
Social support						
Group	0.897 (–1.196 to 2.991)	0.71 (1)	.40	0.682 (–0.859 to 2.223)	0.75 (1)	.39
Time1	–0.23 (–3.207 to 2.747)	0.02 (1)	.88	–0.223 (–3.197 to 2.751)	0.02 (1)	.88
Time2	–2.429 (–4.746 to –0.112)	4.22 (1)	.04	–2.43 (–4.732 to –0.129)	4.28 (1)	.04
Time3	–0.368 (–2.593 to 1.857)	0.11 (1)	.75	–0.373 (–2.607 to 1.861)	0.11 (1)	.74
Group×Time1	0.973 (–2.303 to 4.250)	0.34 (1)	.56	0.966 (–2.300 to 4.231)	0.34 (1)	.56
Group×Time2	3.636 (1.059 to 6.212)	7.65 (1)	.006	3.669 (1.033 to 6.306)	7.44 (1)	.006
Group×Time3	1.74 (–0.584 to 4.065)	2.15 (1)	.14	1.732 (–0.559 to 4.023)	2.20 (1)	.14
Coping modes to confrontation dimension						
Group	0.001 (–1.353 to 1.355)	<0.001 (1)	.10	–0.233 (–1.512 to 1.046)	0.13 (1)	.72
Time1	0.159 (–1.568 to 1.886)	0.03 (1)	.86	0.17 (–1.567 to 1.907)	0.04 (1)	.85
Time2	–0.324 (–1.449 to 0.800)	0.32 (1)	.57	–0.32 (–1.456 to 0.816)	0.30 (1)	.58
Time3	–0.521 (–1.352 to 0.309)	1.51 (1)	.22	–0.523 (–1.365 to 0.318)	1.49 (1)	.22
Group×Time1	0.298 (–1.452 to 2.047)	0.11 (1)	.74	0.287 (–1.471 to 2.045)	0.10 (1)	.75
Group×Time2	1.475 (0.238 to 2.711)	5.46 (1)	.02	1.500 (0.244 to 2.755)	5.48 (1)	.02
Group×Time3	1.691 (0.645 to 2.737)	10.04 (1)	.002	1.675 (0.621 to 2.730)	9.69 (1)	.002
Coping modes to avoidance dimension						
Group	1.886 (0.824 to 2.948)	12.12 (1)	<.001	1.886 (0.824 to 2.948)	12.12 (1)	<.001
Time1	1.391 (0.858 to 1.925)	26.14 (1)	<.001	1.391 (0.858 to 1.925)	26.14 (1)	<.001
Time2	0.774 (0.261 to 1.287)	8.73 (1)	.003	0.774 (0.261 to 1.287)	8.73 (1)	.003
Time3	0.836 (0.553 to 1.118)	33.62 (1)	<.001	0.836 (0.553 to 1.118)	33.62 (1)	<.001
Group×Time1	–1.706 (–2.870 to –0.541)	8.24 (1)	.004	–1.706 (–2.870 to –0.541)	8.24 (1)	.004
Group×Time2	–2.233 (–3.837 to –0.629)	7.45 (1)	.006	–2.233 (–3.837 to –0.629)	7.45 (1)	.006
Group×Time3	–1.8 (–3.277 to –0.323)	5.70 (1)	.02	–1.8 (–3.277 to –0.323)	5.70 (1)	.017
Coping modes acceptance to resignation dimension						
Group	–0.147 (–0.494 to 0.201)	0.69 (1)	.41	0.084 (–0.389 to 0.557)	0.12 (1)	.73
Time1	0.109 (–0.444 to 0.663)	0.15 (1)	.70	0.103 (–0.438 to 0.643)	0.14 (1)	.71
Time2	0.347 (–0.484 to 1.177)	0.67 (1)	.41	0.350 (–0.503 to 1.202)	0.65 (1)	.42
Time3	0.642 (–0.828 to 2.112)	0.73 (1)	.39	0.649 (–0.837 to 2.135)	0.73 (1)	.39
Group×Time1	–0.538 (–1.159 to 0.083)	2.88 (1)	.09	–0.531 (–1.136 to 0.073)	2.97 (1)	.09
Group×Time2	–0.816 (–1.673 to 0.041)	3.48 (1)	.06	–0.855 (–1.743 to 0.033)	3.56 (1)	.06
Group×Time3	–1.103 (–2.688 to 0.482)	1.86 (1)	.17	–1.095 (–2.697 to 0.506)	1.80 (1)	.18

### Treatment Burden

There was a statistically significant difference between the 2 groups in unplanned hospital visits (*Z*=−0.354; *P*=.01), indicating that the intervention group had fewer unplanned hospital visits than the control group (shown Multimedia Appendix 3).

### Results of Qualitative Analysis

#### Participant Basic Information

A total of 9 participants were interviewed. Their ages ranged from 30 to 53 years. Five participants underwent breast-conserving surgery, while 4 underwent total resection. The number of times participants engaged in the intervention sessions ranged from 3 to 5. Interview durations varied between 21 and 50 minutes. The general information of participants interviewed is shown in Multimedia Appendix 4.

#### Participation Experience

Analysis of participants’ evaluations of the peer support intervention for psychosocial adjustment in young- to middle-aged patients with breast cancer revealed 4 key themes: enhancing psychosocial adjustment, serving as a vital source of support, increasing treatment confidence, and promoting positive coping. The qualitative interview results of intervention participants are shown in Table 5.

**Table 5.** Qualitative interview results of intervention participants.

Theme and subtheme	Quotes
Promote psychosocial adjustment	
Diagnosis and treatment adjustment	“I learned about others’ experiences and how they differed from mine. Realizing I wasn’t unique in this made me feel more at ease.” (P1) “At first, I didn’t know anything about wound care or chemo. After joining the program, I started learning from the peers—how to eat properly, how to manage things.” (P3)
Negative emotions adjustment	“Initially, I was quite scared and felt a lot of pressure. After chatting with peer, those worries have largely faded.” (P2) “During the hardest times, chatting with the peers really made me feel so much lighter and more relaxed.” (P7)
Sexuality and body image adjustment	“After chemotherapy, my hair started falling out a lot, so I shaved it off and started wearing a wig. I had already bought the wig right after I attended the third session.” (P5) “I hadn’t thought about sexual intimacy before, but after discussion, I talked to my husband. He was initially unsupportive, but later understood not to pressure me. Following their suggestions, we still kiss, hug, and hold hands.” (P8)
Family role functioning adjustment	“My family cares about me, but we don’t discuss life issues much with peers; I talk more with friends or close confidants.” (P3) “After I got sick, my sister came to take care of me, so things at home are fine. I don’t talk much about family stuff with the peers.” (P4)
Social participation adjustment	“They encouraged me to go out and work instead of staying home, which had a big impact. I’ve been working continuously.” (P8) “Following the advice from the peers, I cut back on pointless socializing. If chatting with friends does not feel right for my current state of mind, I just pause it for now. It makes things a lot less hard on my heart.” (P9)
Serving as a vital source of support	
Informational support	“When I was diagnosed, I didn’t know a tumor meant cancer. Joining the sessions helped me learn a lot about the disease and post-surgery care.” (P4) “Most of them have already had kids, but I haven’t yet, so a lot of people probably haven’t been in my situation. One peer talked about egg freezing, and that really helped me—it gave me useful information. I think that was especially good.” (P6)
Care and encouragement	“They really care about us—they often ask if we’re in pain, if we can eat, if we’re throwing up.” (P1) “They’re very responsible, often asking in the chat group how we’re recovering or doing. Their heartfelt chats helped me through the initial depressive phase.” (P2)
Space for expression	“I became good friends with one of the group members—we chat every day now, even about our everyday lives.” (P7) “The Chat group is great—it gives me a space to communicate and vent. There are things I wouldn’t say to my husband or mother, but I can share them privately with group members.” (P9)
Increasing treatment confidence	
	“Some people have it way worse than me and they’ve lived for over ten years. So I think to myself, my life won’t be short—I’ll definitely be okay and get to see my child grow up.” (P1) “Seeing others recover and return to work normally boosts my confidence. If they can live well, I can strive for it too and feel capable.” (P3)
Promoting positive coping	
	“Initially, I was in a bad mood and reluctant to join, but after attending, the open discussions really reduced my stress. Facing the disease head-on, following the doctor’s treatment plan, and staying committed gives me hope for better days.” (P2) “I was always so scared and kept avoiding it—I cried a lot. But after the first peer meeting, when I heard the peers and nurses talk about it, I felt like there was nothing to be afraid of anymore. Just go ahead with the treatment.” (P9)

## Discussion

### Principal Findings

Young- to middle-aged patients with breast cancer face significant psychosocial challenges, which can impair immune function and worsen prognosis [8,48]. Addressing these issues with effective interventions is crucial—the PNO2PSP notably improved psychosocial adjustment, supported by qualitative interviews. A similar trial by Høybye et al [49] found improvements in helplessness and depression, though without affecting broader mental adjustment or mood disturbance. The difference likely stems from the lack of trained peer supporters and structured interventions in that study [50]. In contrast, this study used various support formats, including peer education and meetings with nurses, ensuring more stable social networks and addressing real concerns. Peer support groups also offered a space for participants to communicate anytime and anywhere, addressing adjustment issues promptly [25,51].

In terms of feasibility, this study recruited participants preoperatively, with a total of 90 patients contacted and 70 (77.8%) consenting to participate. Attrition rates were 12.4% in the control group and 10.5% in the intervention group, primarily occurring during the follow-up phase after the intervention. During the first 8 weeks of the intervention, the overall participation rate in the intervention group was high at 86.3%. This high rate can be attributed to the intervention themes being tailored to the specific psychosocial adjustment challenges faced by young- to middle-aged patients with breast cancer. Additionally, the blended online and offline intervention model reduced logistical and financial burdens, allowing participants to participate from home [52]. The intervention’s feasibility was further enhanced by its design. Participants scheduled for surgery were recruited during their hospital stay, allowing ample time for baseline assessments and the initial offline intervention session, which facilitated participant engagement. Expert consultations and focus group interviews determined the blended intervention format. The initial offline session established connections and trust between peers and participants, while subsequent online sessions minimized the need for hospital visits, increasing participation and motivation. The study established online support groups for participants, where the actual frequency of group discussions exceeded the initially planned schedule, reflecting a high level of participant engagement. This enthusiasm was largely driven by the peer supporters, who regularly followed up with participants in the online support groups, provided necessary information, and responded promptly to participants’ questions. For issues requiring professional expertise beyond the scope of peer supporters, nurses stepped in to provide timely assistance. Furthermore, each online support group included at least 2 peer supporters, ensuring that if 1 was unavailable, the other could respond without delay.

PNO2PSP significantly improved the psychosocial adjustment of young- to middle-aged patients with breast cancer, a finding further supported by the results of qualitative interviews. Similar findings were reported by Høybye et al [49], who conducted a randomized controlled trial of an internet peer support group for patients with breast cancer. However, their study showed improvements in helplessness, confusion, and depression, with no significant effects on other dimensions of mental adjustment to cancer or total mood disturbance. The difference in results likely stems from the previous study’s reliance on mutual support among participants without trained peer supporters [50]. Additionally, the intervention lacked clear themes and fixed schedules, and the online-only format made it difficult for participants to establish a stable social network. In contrast, this study adopted a variety of peer support formats. Peer education involved trained peers sharing their personal experiences and coping modes with the disease. Peer group meetings provided a platform for participants to interact with peer supporters and nurses, offering an opportunity for emotional expression. Peer support groups offered a space for participants to communicate anytime and anywhere, addressing adjustment issues promptly [25,51]. Moreover, the intervention themes in this study were developed based on specific adjustment issues identified through qualitative research, ensuring that they addressed real and relevant concerns faced by young- to middle-aged patients with breast cancer.

At the same time, the analysis of the subdimensions of psychosocial adjustment showed that the intervention improved participants’ scores in the sexual relationship dimension. A previous study found that peer support did not effectively improve sexual relationships among patients with breast cancer [29]. The discrepancy may lie in the fact that previous peer support studies, even when addressing sexuality-related topics, focused only on knowledge dissemination, offering participants limited actionable advice [53]. In this study, through the use of intervention manuals, peer supporters’ experience sharing, and nurses’ education, participants received knowledge related to sexual health. This not only informed participants that they could engage in sexual activities postsurgery but also encouraged them to actively discuss and resume sexual activities [54]. Participants were provided with techniques to alleviate discomfort during sexual intercourse, and peer supporters shared practical ways to maintain intimacy beyond intercourse, such as kissing and hugging [55]. These actionable strategies significantly improved sexual relationships. Influenced by traditional Chinese culture, sexual health education has long been a challenge in educating patients with breast cancer [14].

However, because peer supporters in this study were all women of similar age and with similar experiences, participants could freely discuss sexuality-related issues without feeling embarrassed [55]. Therefore, peer support could be a viable approach for improving sexual health among patients with breast cancer in the future. The intervention also improved participants’ psychological distress scores. Interviews revealed that participants believed peer support helped alleviate negative emotions, reducing their suffering, anxiety, and fear, which aligns with the findings of previous studies. A previous systematic review on peer support for patients with cancer indicated that peer support interventions effectively reduce depression and anxiety [56]. In many Asian societies, cultural norms surrounding emotional expression, illness disclosure, and discussion of family matters may influence how openly patients communicate within peer support groups. This cultural context may partly explain the limited effects observed in family-related dimensions of psychosocial adjustment [57]. However, there was no statistically significant improvement in the social relationship dimension, possibly due to the short follow-up period. At the end of follow-up, many participants were still in treatment and had not yet resumed social reintegration. Nonetheless, qualitative interviews showed that participants believed peer support could promote their occupational and social adjustment.

The findings revealed that the intervention could enhance the levels of social support, consistent with the results of previous studies [50,58]. In the design of this study, peer support provided participants with informational, emotional, and appraisal support. When participants had questions about their disease or treatment, peer support offered informational support and guided them to seek professional support, family support, and other social support resources [25]. When participants experienced negative emotions due to illness and treatment, peer supporters provided emotional support through listening, comforting, and encouraging, which improved individuals’ perceived levels of social support [59]. Additionally, appraisal support helped participants reevaluate the support resources they possessed, enhancing their subjective perception of social support [60]. This program also facilitated the adoption of problem-focused coping strategies while reducing the use of avoidance coping strategies. The interview results with participants supported this finding. A prior study on peer support for adolescent patients with cancer [61] yielded similar results, where participants who received peer support adopted more coping strategies 3 months postsurgery. The reasons for these outcomes include peer supporters sharing their own experiences in addressing psychosocial issues, providing participants with reference points that reduced their fear and avoidance of disease-related challenges [62]. Through the intervention, participants’ attitudes toward their illness underwent a significant transformation—they became more willing to confront cancer directly, ceased avoiding cancer-related issues, actively sought ways to resolve problems, and approached their condition with greater positivity and optimism [63].

Our qualitative findings supported that the intervention contributed to increasing treatment confidence among participants. However, the intervention did not produce a statistically significant improvement in self-efficacy among young- to middle-aged patients with breast cancer. Similarly, a prior study [64] reported that a 6-month peer counseling intervention for patients with breast cancer failed to significantly enhance their self-efficacy. A systematic review on peer support interventions for patients with cancer also found that peer support interventions did not improve participants’ self-efficacy [56]. However, a previous study demonstrated that a 6-week dyadic peer intervention improved patients with breast cancer self-management self-efficacy among patients with breast cancer [65]. The potential reasons for this discrepancy may be related to the measurement tool used in this study. The GSES used may lack the sensitivity needed to detect changes in the self-efficacy specific to coping with illness [66]. Mechanistically, self-efficacy relies on structured mastery experiences, whereas our program emphasized peer empathy and informational support over skill-based tasks. Furthermore, the robust external support from nurses and peers may have improved adjustment but temporarily bypassed the need for participants to develop independent self-reliance. Alternatively, peer support may primarily enhance disease-specific self-efficacy, which has a limited impact on overall self-efficacy [65]. Another possible explanation is that self-efficacy tends to be a stable personal attribute that is difficult to change over a short period [56].

The study results also showed that the intervention significantly reduced the number of unplanned hospital visits among participants. Similarly, a previous study [67] found that peer support interventions for patients after coronary artery bypass surgery led to reduced health care usage 3 months postsurgery, with fewer visits to general practitioners and emergency departments. This reduction is likely attributable to the synergy between peer supporters, who provided immediate experiential guidance for common postdischarge concerns, and intervention nurses, who offered professional triage and symptom management advice. This collaborative approach effectively addressed minor complications that might otherwise have triggered unnecessary hospital visits. This reduction in hospital readmissions may help alleviate patients’ financial burden and reduce the strain on health care systems. Although the result confirmed the robustness of this finding, the reliance on self-reported health care usage data remains a limitation. Future large-scale trials should integrate clinical records with a formal economic framework to conduct a comprehensive cost-utility analysis.

### Limitations

This study has certain limitations. This study used a single-center CRCT with a limited sample size (N=70), which may restrict the generalizability of the findings. Furthermore, as the primary outcomes were based on self-reported scales, this lack of blinding may have introduced potential self-report bias. In addition, participant attrition during the follow-up period may further limit the robustness and generalizability of the results. Future research should consider multicenter, large-sample trials to further evaluate the effectiveness of peer support. Although this study focused on early postoperative intervention and follow-up, extending the intervention period in future research may further enhance participants’ psychosocial adjustment during the recovery phase. Furthermore, this study only collected self-reported patient expenditures without performing a comprehensive economic analysis.

### Conclusion

Peer support represents a potentially more cost-effective and sustainable form of support. This study developed a PNO2PSP for psychosocial adjustment in young- to middle-aged patients with breast cancer, guided by a complex intervention framework. The PNO2PSP demonstrates strong scientific validity and feasibility. It effectively improves the psychosocial adjustment levels of young- to middle-aged patients with breast cancer, enhances social support, and promotes positive coping modes. By continuously training peer supporters and conducting peer support interventions, the psychosocial adjustment of young- to middle-aged patients with breast cancer can be further promoted, and this PNO2PSP could be standardized and widely implemented in clinical practice in the future.

## Supplementary material

10.2196/86097 Multimedia Appendix 1 Schedule and details of the peer-led, nurse-involved, blended online and offline peer support intervention program (PNO2PSP).

10.2196/86097 Multimedia Appendix 2 Results of the intervention effect evaluation of the subdimensions of psychosocial adjustment.

10.2196/86097 Multimedia Appendix 3 Comparison of secondary outcomes between the intervention group and the control group at different time points.

10.2196/86097 Multimedia Appendix 4 General information of participants interviewed (N=9).

10.2196/86097 Checklist 1 CONSORT 2010 checklist of information to include when reporting a cluster randomized trial.

## Abbreviations

CRCT: cluster randomized controlled trial
DE: design effect
GEE: generalized estimating equation
GSES: General Self-Efficacy Scale
MCMQ: Medical Coping Modes Questionnaire
PAIS: Psychosocial Adjustment to Illness Scale
PNO2PSP: peer-led, nurse-involved, blended online and offline peer support intervention program
SSRS: Social Support Rate Scale
