# Supplementary material for: A Peer-Led, Nurse-Involved Blended Online and Offline Peer Support Program (PNO2PSP) for Psychosocial Adjustment in Young- to Middle-Aged Patients With Breast Cancer: Cluster Randomized Clinical Trial
Source: J Med Internet Res. 2026 Apr 17;28:e86097. doi: 10.2196/86097 (PMC13089621; doi:10.2196/86097)
Supplement: Multimedia Appendix 1 [file jmir-v28-e86097-s001.docx]

**Table S1 Schedule and details of the PNO2PSP**

| **Session** | **Format** | **Theme** | **Format** | **Content** | **Intervention provider** |
| --- | --- | --- | --- | --- | --- |
| First session(before the surgery) | Offline | breast cancer diagnosis and treatment adjustment  (Offline) | peer experience sharing | Peers share their experiences and coping strategies related to their breast cancer diagnosis and treatment | Peers |
|  |  |  | peer-led group discussions | **Listening and sharing**: participants introduce themselves and share their experiences with breast cancer diagnosis and treatment, and discuss the challenges they have encountered in coping with the disease.  **Emotional response**: peers expressing empathy towards the issues raised by participants, comforting them about the normal issues that every patient has to confront, and sharing their own approaches when encountering similar problems.  **Appraisal and feedback:** peers recognize and affirm positive changes made by participants in adjusting to the illness. Using personal experiences as examples, peers inform participants that proactive coping strategies can lead to better adaptation to the disease, overcoming challenges it presents, and enhancing their confidence. | Peers  Intervention nurses |
|  |  |  | group health education | Providing participants with pertinent information on breast cancer diagnosis and treatment, including primary diagnostic methods, treatment modalities, prognosis, and the time and resources involved in treatment. | Intervention nurses |
| Second session (one week post-surgery) | Online | negative emotion adjustment  (Online)) | peer experience sharing | Peers share their own experiences of previously encountered negative emotions and coping strategies for managing negative emotions. | peers |
|  |  |  | peer-led group discussions | **Listening and sharing**: participants introduce themselves and share their experiences with negative emotions, and discuss the challenges they have encountered in coping with negative emotions.  **Emotional response & Appraisal and feedback:** As above, adjusting flexibly based on the context and theme. | Peers  Intervention nurses |
|  |  |  | group health education | Providing participants with relevant approaches to alleviate negative emotions, primarily including exercise, self-disclosure, relaxation training, breathing exercises, music therapy, etc. | Intervention nurses |
| Third session (three weeks post-surgery) | Online | sexuality and body image adjustment | peer experience sharing | Peers share their own experiences of previously encountered sexuality and body image and coping strategies for managing sexuality and body image. | peers |
|  |  |  | peer-led group discussions | **Listening and sharing**: participants introduce themselves and share their experiences with sexuality and body image, and discuss the challenges they have encountered in coping with sexuality and body image.  **Emotional response & Appraisal and feedback:** As above, adjusting flexibly based on the context and theme. | Peers  Intervention nurses |
|  |  |  | group health education | Offering participants information on the alleviation of concerns related to sexuality and body image, primarily addressing issues such as sexual dysfunction, prosthesis wearing, wound and scar care, weight management, makeup and skincare, and hair loss caused by chemotherapy, etc. | Intervention nurses |
| Fourth session (five weeks post-surgery) | Online | family role adjustment | peer experience sharing | Peers share their own experiences of previously encountered challenges in changes in family role functions and strategies for coping with adjustments in family roles. | peers |
|  |  |  | peer-led group discussions | **Listening and sharing**: participants introduce themselves and share their experiences with changes in family role functions, and discuss the challenges they have encountered in coping with adjustments in family roles.  **Emotional response & Appraisal and feedback:** As above, adjusting flexibly based on the context and theme. | Peers  Intervention nurses |
|  |  |  | group health education | Providing nursing and management strategies for various postoperative adjuvant therapies, including the management of side effects from chemotherapy, radiation therapy, hormone therapy, and targeted therapy, as well as dietary and exercise recommendations for different treatment phases. | Intervention nurses |
| Fifth session (seven weeks post-surgery) | Online | social participation adjustment. | peer experience sharing | Peers share their own experiences of previously encountered challenges in changes in social participation and strategies for coping with adjustments in social participation. | peers |
|  |  |  | peer-led group discussions | **Listening and sharing**: participants introduce themselves and share their experiences with changes in social role functions, and discuss the challenges they have encountered in coping with social participation.  **Emotional response & Appraisal and feedback:** As above, adjusting flexibly based on the context and theme. | Peers  Intervention nurses |
| Every week | Online |  | online Peer Chat Groups | **Online discussions:** Participants share the adaptive challenges they face in their daily lives, and peers provide informational, emotional, and appraisal support. Intervention nurses offer additional informational support as needed.  **Initiative format:** Participants can ask questions and start discussions at any time. If there are no participant queries, peers will initiate conversations, ensuring that online interactions occur at least twice a week. | Peers  Intervention nurses |
