# Supplementary material for: A Peer-Led, Nurse-Involved Blended Online and Offline Peer Support Program (PNO2PSP) for Psychosocial Adjustment in Young- to Middle-Aged Patients With Breast Cancer: Cluster Randomized Clinical Trial
Source: J Med Internet Res. 2026 Apr 17;28:e86097. doi: 10.2196/86097 (PMC13089621; doi:10.2196/86097)
Supplement: Multimedia Appendix 2 [file jmir-v28-e86097-s002.docx]

**Figure S1.** Results of the intervention effect evaluation: subdimensions of psychosocial adjustment.


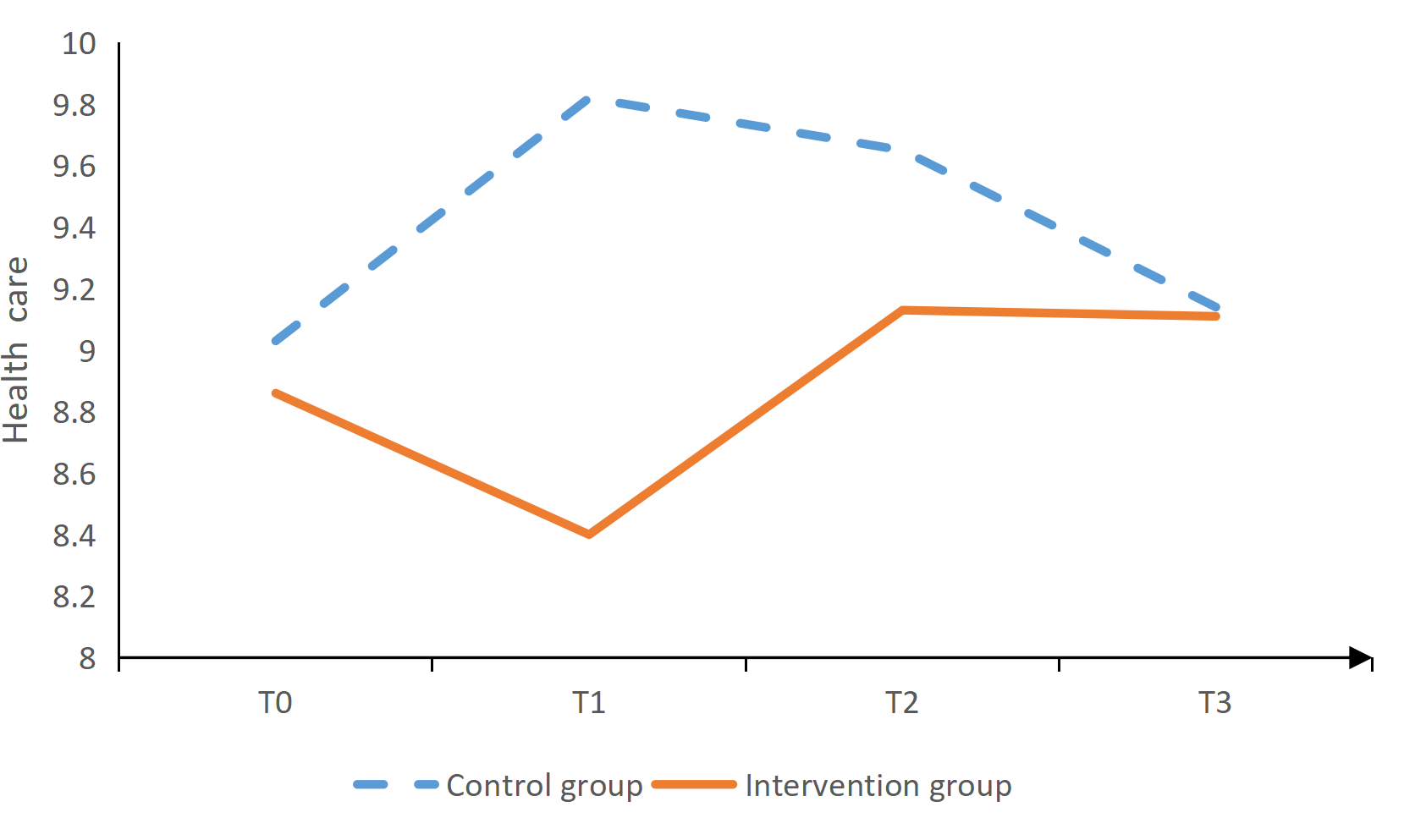


1. Health care


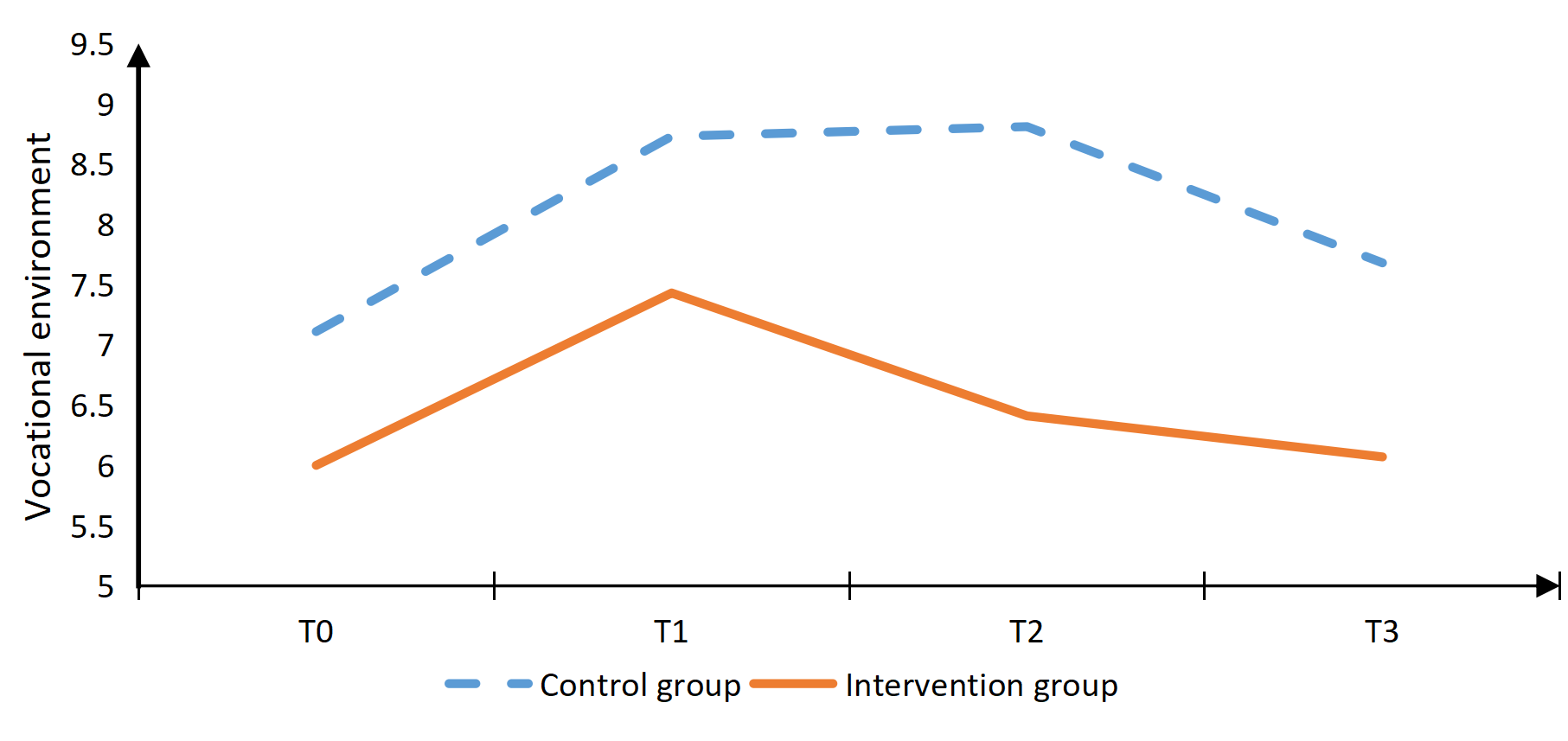


1. Vocational environment


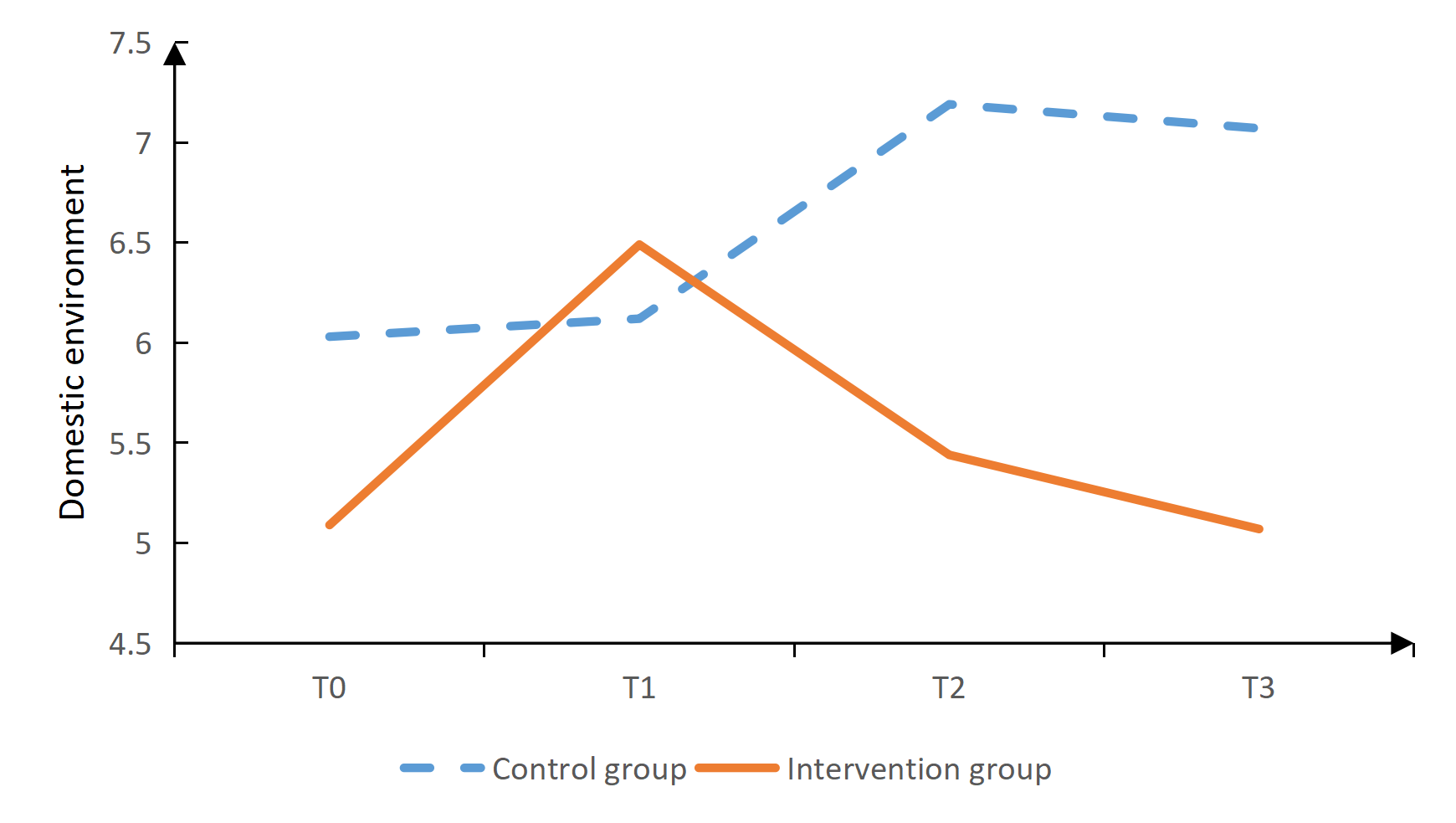


1. Domestic environment


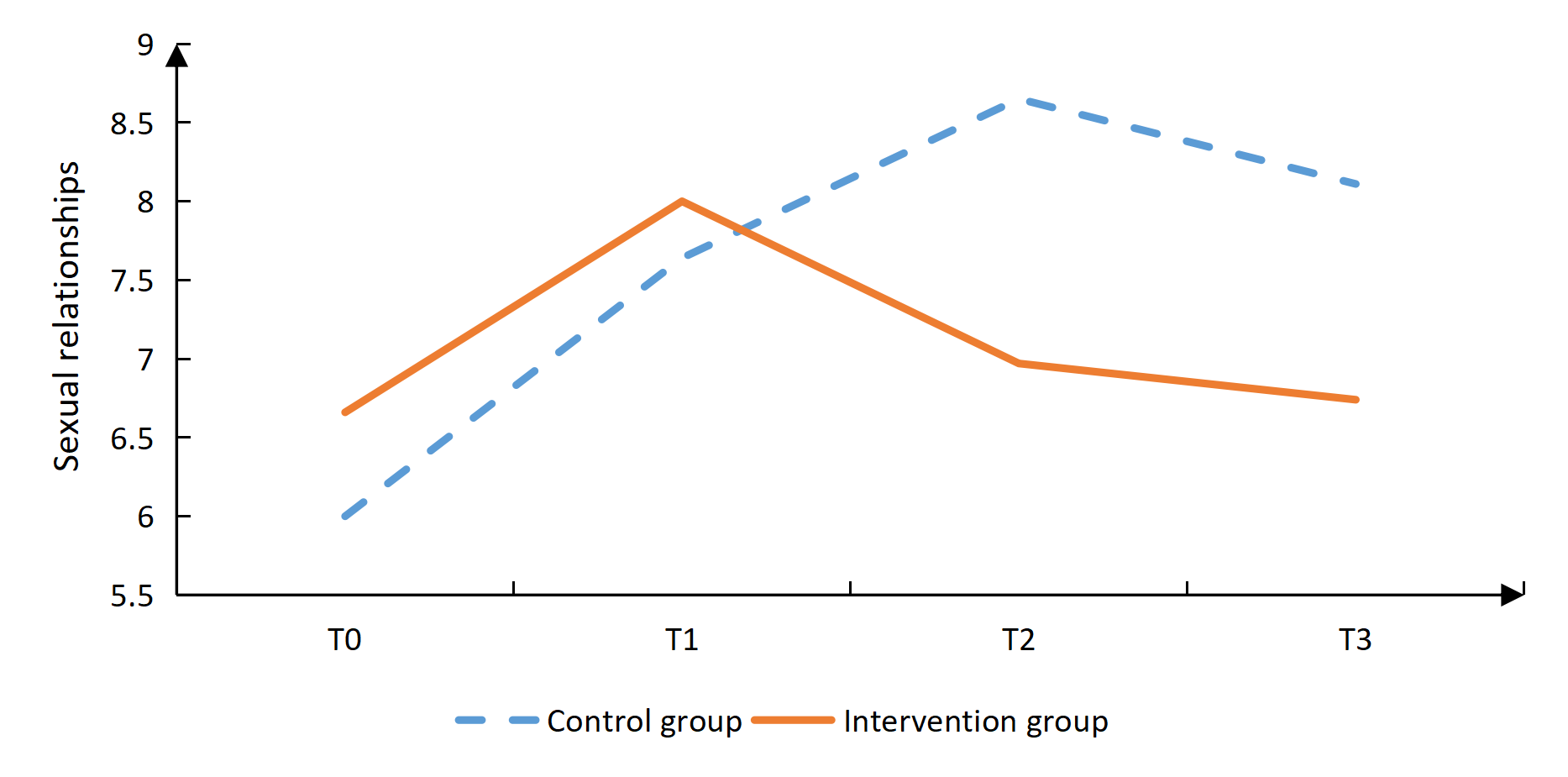


1. Sexual relationships


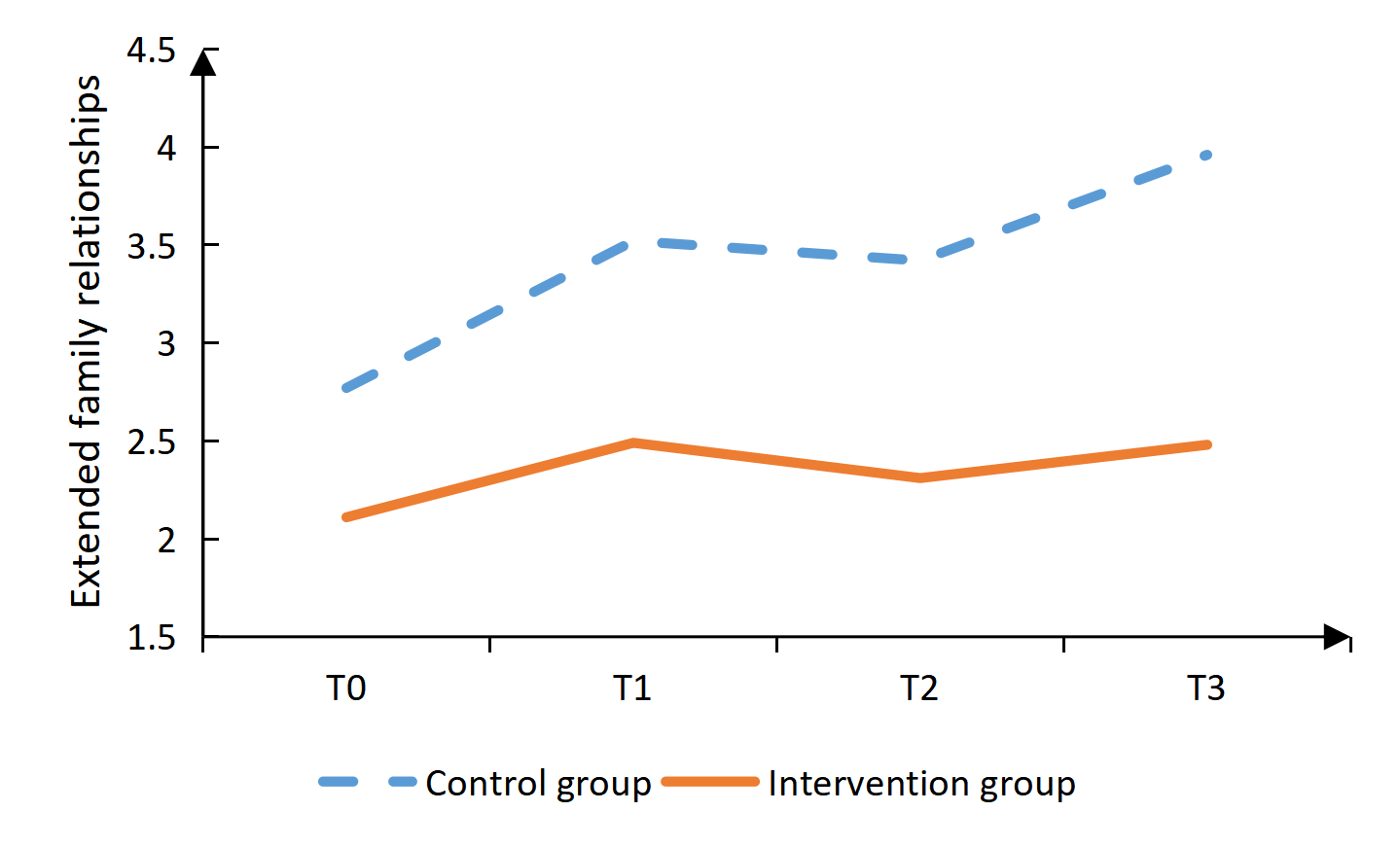


1. Expand family relationships


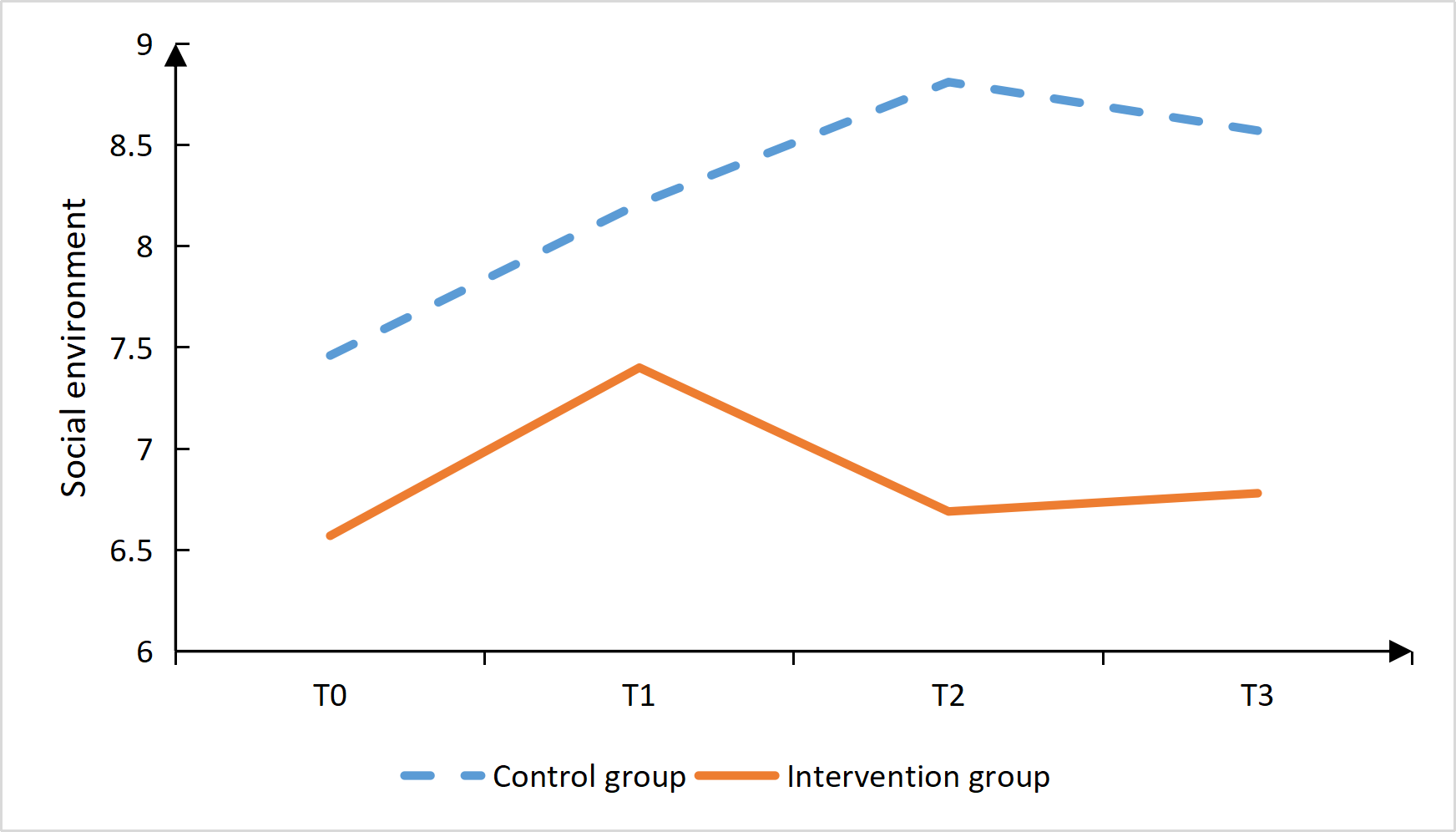


1. Social environment


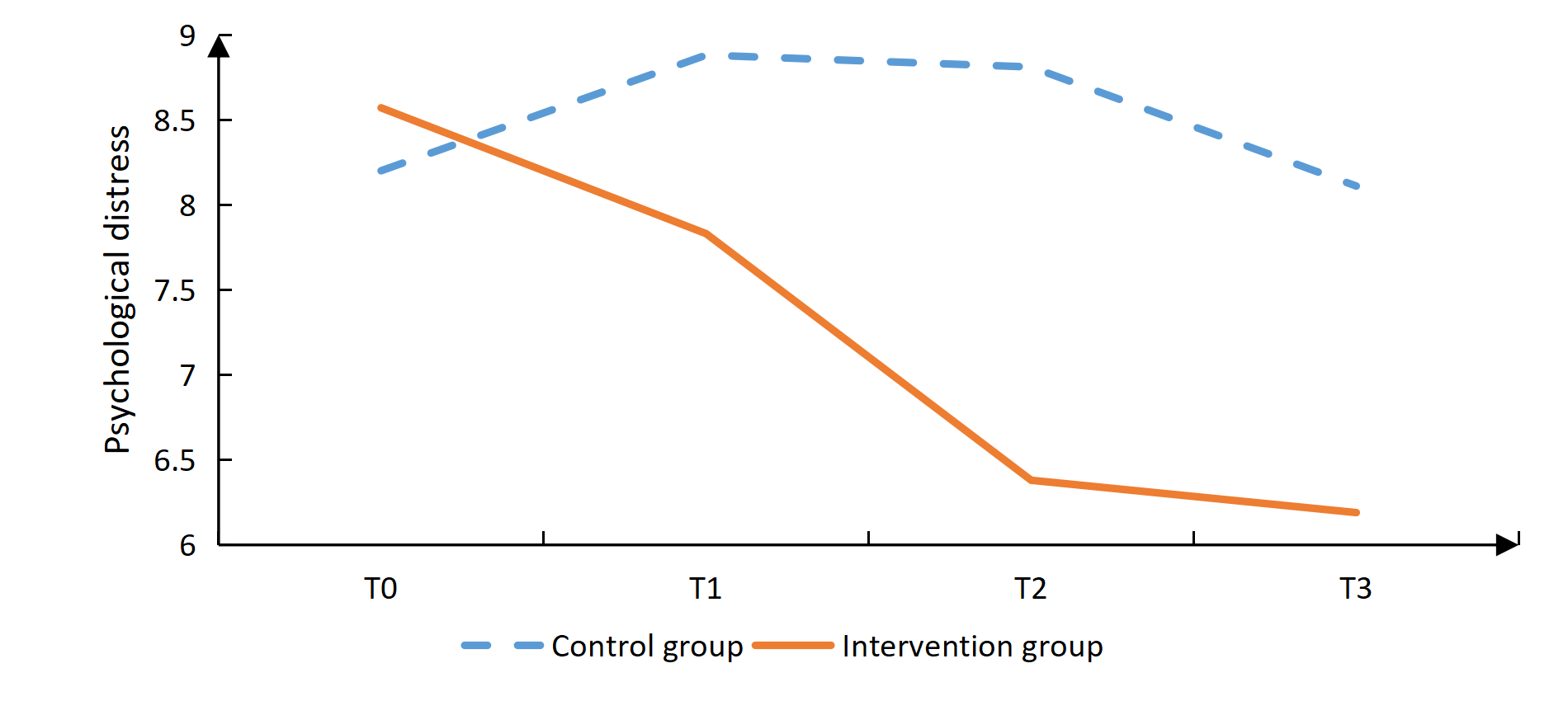


1. Psychological distress
