# Supplementary material for: A Peer-Led, Nurse-Involved Blended Online and Offline Peer Support Program (PNO2PSP) for Psychosocial Adjustment in Young- to Middle-Aged Patients With Breast Cancer: Cluster Randomized Clinical Trial
Source: J Med Internet Res. 2026 Apr 17;28:e86097. doi: 10.2196/86097 (PMC13089621; doi:10.2196/86097)
Supplement: Multimedia Appendix 3 [file jmir-v28-e86097-s003.docx]

| **Item** | **Group** | **N** | ***M*(***Q*_25_,*Q*_75_**)** | ***Z*** | ***P*** |
| --- | --- | --- | --- | --- | --- |
| Treatment Cost (thousand yuan) | Control Group | n=20 | 100(50, 150) | -0.375 | 0.714 |
|  | Intervention Group | n=24 | 66(50, 138) |  |  |
| Unplanned hospital visits | Control Group | n=20 | 1.50(0, 5.75) | -2.529 | **0.011** |
|  | Intervention Group | n=24 | 0(0, 1) |  |  |
| Outpatient Visits | Control Group | n=20 | 15(8, 30) | -0.354 | 0.730 |
|  | Intervention Group | n=24 | 11(8, 20) |  |  |
| Total Hospitalization Times | Control Group | n=20 | 5(1, 7) |  |  |
|  | Intervention Group | n=24 | 5(2, 7) | -0.446 | 0.662 |
