# Supplementary material for: A Peer-Led, Nurse-Involved Blended Online and Offline Peer Support Program (PNO2PSP) for Psychosocial Adjustment in Young- to Middle-Aged Patients With Breast Cancer: Cluster Randomized Clinical Trial
Source: J Med Internet Res. 2026 Apr 17;28:e86097. doi: 10.2196/86097 (PMC13089621; doi:10.2196/86097)
Supplement: Multimedia Appendix 4 [file jmir-v28-e86097-s004.docx]

| Number | Age  (years) | Number of children | Surgical type | Times of sessions participation | Interview duration (minutes) |
| --- | --- | --- | --- | --- | --- |
| P1 | 38 | 4 | Breast-conserving surgery | 4 | 37 |
| P2 | 53 | 2 | Breast-conserving surgery | 3 | 25 |
| P3 | 36 | 2 | Total Resection | 4 | 44 |
| P4 | 41 | 3 | Breast-conserving surgery | 4 | 21 |
| P5 | 49 | 1 | Total Resection | 5 | 28 |
| P6 | 51 | 1 | Breast-conserving surgery | 5 | 42 |
| P7 | 48 | 1 | Total Resection | 4 | 28 |
| P8 | 30 | 0 | Breast-conserving surgery | 5 | 35 |
| P9 | 31 | 0 | Breast-conserving surgery | 5 | 50 |
